# PAF Triggered Pyroptotic NETosis Aggravates Myocardial Ischemia/Reperfusion Injury

**DOI:** 10.1002/advs.202519140

**Published:** 2026-02-25

**Authors:** Jiawei Wu, Shule Zhang, Ruofan Du, Lina Kang, Guodong Zhao, Xue Bao, Haochi Yang, Ziqing Xie, Tianyu He, Huiyong Sun, Haiping Hao, Lijuan Cao

**Affiliations:** ^1^ State Key Laboratory of Natural Medicines China Pharmaceutical University Nanjing P. R. China; ^2^ Jiangsu Provincial Key Laboratory of Targetome and Innovative Drugs Institute of Innovative Drug China Pharmaceutical University Nanjing P. R. China; ^3^ Department of Cardiology Nanjing Drum Tower Hospital Affiliated to Nanjing University Medical School Nanjing China; ^4^ Department of Gastroenterology Kunshan Hospital Affiliated to Jiangsu University Kunshan China

**Keywords:** dapagliflozin, myocardial ischemia‐reperfusion injury, NETosis, platelet activating factor

## Abstract

Myocardial ischemia‐reperfusion (MI/R) injury remains a critical challenge in cardiovascular therapeutics, with metabolic‐inflammatory signaling axis emerging as a critical mediator of pathological outcomes. Yet, the specific metabolic pathways interplay with inflammation to exacerbate MI/R injury remain poorly defined. Here we verify that NETosis of neutrophils is an initiative and causal factor in driving MI/R injury, specifically, platelet activating factor (PAF) secreted by cardiomyocytes during MI/R, drives neutrophil extracellular traps (NETs) formation and subsequent NETosis. Increased expression of PAF synthesis enzyme PLA2G6 explains excessive production of PAF. PAF‐induced NETosis requires gasdermin D (GSDMD) mediated pore‐forming to facilitate NETs extrusion. Both inhibiting NETs and PAF synthesis significantly alleviate MI/R injury. We further identify dapagliflozin as a potent NETosis inhibitor that protects mice from MI/R injury in a sodium‐glucose co‐transporter 2 (SGLT2)‐independent manner, which targets neutrophil gelatinase‐associated lipocalin‐2 (LCN2). Notably, increased serum PAF concentration in acute myocardial infarction patients with percutaneous coronary intervention was positively correlated with NETosis and myocardial injury indexes. Of interest, patients receiving dapagliflozin exhibited attenuated myocardial injury in comparison to those without dapagliflozin. Collectively, our study demonstrates PAF serves as a danger signal in triggering NETosis in early MI/R injury, and manipulating PAF‐NETosis signal by dapagliflozin or LCN2 inhibitor might be effective in combating MI/R injury.

## Graphical abstract

Platelet activating factor (PAF) secreted by cardiomyocytes during MI/R, drives NETs formation and subsequent NETosis. PAF/NETosis signaling activation is an initiative and causal factor in driving MI/R injury. This study identifies dapagliflozin as a potent NETosis inhibitor, and demonstrates that manipulating PAF‐NETosis signal by dapagliflozin or LCN2 inhibitor might be effective in combating MI/R injury.

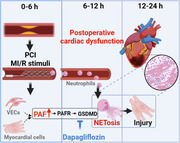

## 1 Introduction

Acute myocardial infarction, characterized by reduced myocardial perfusion and cardiac cell death, is one of the leading causes of mortality [1]. Revascularization therapy can sufficiently restore blood flow in ischemic myocardial tissues and thus has become standard treatment for patients. After reconstruction of blood vessels or treatment with thrombolytic drugs, reperfusion injury inevitably leads to secondary myocardial injury [2]. About 22% patients with timely reperfusion will eventually develop heart failure and even death [3]. Numerous studies have been performed to understand the pathophysiological alterations during reperfusion, including oxidative stress, activation of regulated cell death pathways (e.g., apoptosis, pyroptosis, ferroptosis, and necroptosis), pH shifts, and sterile inflammation. However, it remains largely unclear about how these mechanisms are integrated and finally cause cardiomyocyte death, and few of these findings has been successfully translated to therapeutic options [4, 5, 6, 7]. Moreover, the time window of ischemia reperfusion injury is narrow, and the infarct size, once formed, is irreversible, which may explain why previous findings focused on the later event of myocardial ischemia reperfusion (MI/R) injury are hard to be translatable to effective clinical therapy. Therefore, it is of paramount importance to identify the intrinsic factors and mechanisms underlying the early onset of MI/R injury.

MI/R injury is characterized by a cascade of immunoinflammatory reactions which is supposed to eliminate cell debris and pathogens, but excessive inflammatory activation may cause damage to myocardial tissues [8]. Neutrophil infiltration into reperfused myocardium is central to this pathology, serving as both primary effectors and amplifiers of inflammatory injury by expansion of nucleus and cellular granules to form neutrophil extracellular traps (NETs) [9, 10, 11]. However, little is known about how NETosis is initiated and how NETs regulate immunity and cardiomyocyte death during the ischemia reperfusion. Cytokines like IL‐1β, TNF‐α, IL‐8, and ROS have been considered as the major causes of NETosis in ischemic pathologies [10]. However, in our study, we found that cytokine production lagged behind NETs formation, suggesting that other early events might be responsible for initiating NETs formation. A recent study has indicated that platelet‐neutrophil aggregates enhance NETosis and ROS production, creating a feedforward loop of inflammation during myocardial injury [12]. Yet, how platelets and neutrophils are interplayed and if there is potential mechanical commonality of platelet and neutrophil activation remains unclear. Preclinical studies demonstrate that interventions using DNase I or Cl‐amidine hydrochloride attenuate microvascular obstruction and preserve cardiac function in murine models [13, 14], however, targeting neutrophil‐mediated inflammation remains a translational priority for that the specific pathway or key node that engaging tissue damage effect of neutrophil is largely undefined.

In this study, we systematically delineated the dynamic interplay between immuno‐metabolic signatures and regulated cell death modalities during MI/R injury, with the primary objective of identifying early molecular determinants governing the pathological progression of heart failure. We observed a drastic infiltration of neutrophils in cardiac tissue at an early stage of 3 h and peaked at 12 h after reperfusion, concomitant with NETosis activation at 6 h. Depletion of neutrophils using neutralizing antibody, and inhibition of NETosis by DNase I or Cl‐amidine hydrochloride efficiently blocked NETs formation and alleviated inflammation in MI/R mice. In particular, NETs formation was activated by conditional medium from hypoxia/reoxygenation (H/R)‐conditioned cardiomyocytes. By screening a panel of endogenous metabolites, we identified platelet activating factor (PAF) as a strong trigger of NETosis, which is also elevated during MI/R. The pathological PAF accumulation, mediated by enhanced PLA2G6 enzymatic activity, drives excessive NETosis through a gasdermin D (GSDMD)‐dependent pathway. Finally, we identified dapagliflozin as a potential NETosis inhibitor which is effective for MI/R treatment. Notably, we identified neutrophil gelatinase‐associated lipocalin‐2 (LCN2) as a potential target of dapagliflozin in alleviating MI/R injury. This work provides novel mechanistic insights into early immunometabolic crosstalk in MI/R injury, elucidating how cardiomyocyte‐derived metabolic signals orchestrate neutrophil activation cascades that exacerbate myocardial damage. Our study positions the PAF‐NETosis axis as a promising therapeutic target for retarding MI/R injury.

## 2 Results

### 2.1 Neutrophil Infiltration Is an Early Onset of MI/R

**FIGURE 1:**
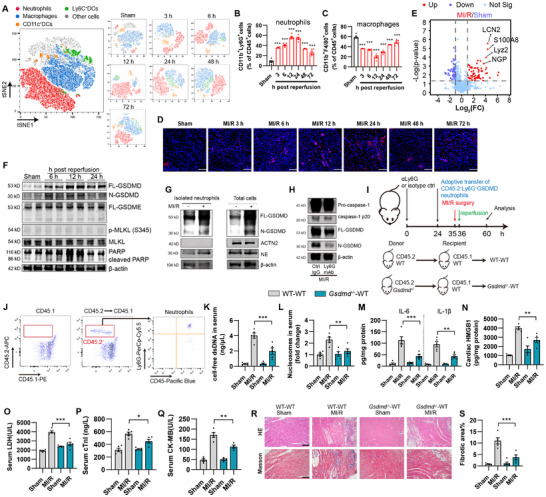
Neutrophils contribute to cardiac injury and cell death during MI/R injury. The left anterior descending coronary artery (LCA) of mice were ligated for 60 min to induce ischemia, then the slipknot is released for indicated time to induce reperfusion (*n* = 5, otherwise indicated). (A–C) Flow cytometry analysis of immune cell population (Neutrophils, CD45^+^CD11b^+^Ly6G^+^; macrophages, CD45^+^CD11b^+^F4/80^+^; Ly6C^+^ monocytes, CD45^+^Ly6C^+^CD11b^+^F4/80^−^Ly6G^−^; myeloid DCs, CD45^+^CD11b^+^CD11c^−^Ly6C^−^) in cardiac tissues at indicated time points. T‐SNE color‐coded maps according to manually gated cell clusters of merged groups (Left panel) and each time points (right panel) (A), percentage of neutrophils (B), and macrophages (C) were shown (*n* = 5). ^***^ *p* < 0.001 compared to the sham group. (D) Representative images of heart section with staining for neutrophil elastase (red) and Hoechst (blue). Scale bar, 50 µm. (E) Volcano plot of proteomics analysis showing protein level changes after MI/R injury. Fold change > 2 or < 0.05 with *p*‐value < 0.05 were highlighted as red or blue, respectively. (F) Immunoblots of GSDMD, GSDME, MLKL, phosphorylated MLKL, and PARP, with β‐actin as loading control. (G) Mice were subjected into LAD ligation for 1 h, followed by reperfusion for 12 h. Cardiac tissues were digested to generate cell suspensions (total cells). Neutrophils were isolated using anti‐Ly6G magnetic beads (isolated neutrophils). GSDMD activation was determined by western blot. (H) Immunoblots of GSDMD and caspase‐1, with β‐actin as loading control. (I) Experimental design of adoptive transfer. (J) Flow cytometry analysis confirms the existence of peripheral CD45.2^+^Ly6G^+^ neutrophils 12 h after adoptive transfer. (K,L) Cell‐free dsDNA (K) and MPO‐DNA nucleosome quantification (L) in serum. (M,N) Cardiac concentration of IL‐6, IL‐1β (M), and HMGB1 (N) determined by ELISA. (O) Serum LDH level. (P) Serum cTnI level. (Q) Serum CK‐MB level. (R) Representative images of HE staining (upper panel) and Masson's trichrome staining (lower panel) of a heart section. Scale bar, 100 µm. (S) Percentage of fibrotic area. n = 5 mice per group. Data are shown as mean±SEM. ^**^ *p* < 0.01, ^***^ *p* < 0.001.

Emerging evidence has showed that sterile inflammatory responses play critical roles in myocardial reperfusion injury during primary percutaneous coronary intervention (PPCI) in acute myocardial infarction (AMI) patients, leading to AMI patients undergoing postoperative cardiac dysfunction and advanced heart failure, but the intertwining of immunocytes infiltration and cardiomyocyte injury remains largely unclear. To gain better insight into the dynamic regulation of inflammation and cardiomyocyte injury in the progression of MI/R, we generated a mouse model of MI/R by ligation of the left anterior descending coronary artery for 60 min followed by reperfusion for series times of 3 to 72 h. MI/R caused prominent cardiac injury at 12 h and fibrosis at 24 h after reperfusion as evidenced by histological assessment, TUNEL staining, Masson staining, and TTC staining (Figure S1A–E). Notably, myocardial IL‐6 was increased early at 6 h post reperfusion, suggesting that the innate immune response is an early onset of cardiac injury (Figure S1F–H). The infiltration of innate immune cells to the injured tissue is an adaptive mechanism to remove the debris for tissue repair. We thus sought to determine the exact type of infiltrated immune cells at a very early stage of tissue injury. To this end, we investigate a panel of immune cell subpopulations, including dendric cells, monocytes, macrophages, and neutrophils that may infiltrate in myocardial tissue (Figure S2). We found that immune cells in the myocardial tissue of MI/R mice were dominated by neutrophils (CD45^+^CD11b^+^Ly6G^+^), which rapidly infiltrated into the myocardial tissue 3 h after reperfusion in MI/R mice, while macrophages showed an opposite variation trend (Figure 1A–C). Immunofluorescence staining of the neutrophil marker Neutrophil Elastase (NE) also showed the early infiltration of neutrophils in myocardial tissues (Figure 1D). Neutrophil‐associated proteins such as LCN2, S100A8, NGP, and Lyz2 were among the most significantly changed proteins, as indicated by a label‐free proteomics analysis of differential proteins enriched in myocardial tissues of MI/R vs. sham mice (Figure 1E; Table S1). As a further validation, we used Ly6G neutralizing antibody to specific deplete neutrophils before the induction of MI/R injury in mice; depletion efficiency was confirmed by flow cytometry analysis of neutrophils in both peripheral blood and cardiac tissues (Figure S3A). Ly6G neutralizing antibody‐treated MI/R mice showed a significant reduction in infarct size, as evidenced by Evans Blue/TTC double staining and less cell death indicated by TUNEL staining (Figure S3B–E). Inflammatory cytokines such as IL‐1β, IL‐6, and HMGB1 were also significantly reduced, as well as serum cTnI and CK‐MB levels indicating cardiac function (Figure S3F–J), suggesting the protective effect of neutrophil depletion on myocardial injury. These results indicate that neutrophil infiltration is an early onset and causal hallmark of MI/R.

### 2.2 Neutrophils Exacerbate MI/R Dependent of GSDMD‐Mediated NETs Formation

Since neutrophil infiltration is an early onset of MI/R injury, we reasoned it may directly aggravate myocardial cell death. To probe cardiac injury related cell death pathways, we analyzed the activation of different cell death pathways including apoptosis (caspase‐3, ‐8, ‐9, PARP), necrosis (p‐MLKL), inflammatory cell death (caspase‐11, ‐1, GSDMD, GSDME), ferroptosis (Fe^2+^ concentration and GSH‐Px activity), copper related cell death (copper concentration), and NETosis (cardiac NE, serum cell‐free dsDNA, and MPO‐DNA complex). Notably, inflammatory caspase‐1 and neutrophil elastase (NE) were activated as early as 6 h post MI/R surgery; apoptosis signal was also instigated as indicated by caspase‐3 and ‐9 activation, while ferroptosis and curroptosis were less detected (Figure S4A–L). In particular, GSDMD was activated prior to massive activation of apoptosis executor PARP as indicated by immunoblots (Figure 1F). We then pretreated mice with caspase‐1, MLKL, NE, and caspase‐3 inhibitor (vx‐765, nec‐1, sivelestat, and z‐DEVD‐FMK, respectively) to evaluate the impact of pyroptosis, necrosis, NETosis, and apoptosis on myocardial infarction in MI/R mice (Figure S4M). We found that mice pretreated with vx‐765 and sivelestat were insensitive to MI/R induced myocardial infarction, as evidenced by histological assessment, Masson staining, TTC staining, as well as serum LDH (Figure S4N–R). Moreover, we validated that MI/R induced myocardial injury was dependent on GSDMD, as indicated by myocardial cytokines, serum LDH, cTnI, CK‐MB, histological assessment, Masson, and TTC staining (Figure S5A–G). *Wild‐type* (WT) mice exhibited with marked NETs formation in myocardial tissue after MI/R, which was not witnessed in *Gsdmd^−/−^* mice as indicated by citrullinated histone H3 (H3Cit) and myeloperoxidase (MPO) staining, serum cell‐free dsDNA (cfDNA), and MPO‐DNA nucleosomes, with no change of infiltrated neutrophil proportion (Figure S5H–L). These results support that in the condition of MI/R, neutrophils infiltrated into myocardial tissue undergo GSDMD‐dependent NETosis to aggravate myocardial injury.

To address whether GSDMD was activated in neutrophils in vivo, we isolated neutrophils in cardiac tissues 24 h after MI/R surgery, and GSDMD activation was determined by immunoblots. As expected, neutrophils isolated from MI/R mice showed obvious GSDMD cleavage, which would be retracted by Ly6G mAb (Figure 1G,H). To further prove GSDMD function in neutrophils activation and NETs formation during MI/R injury in vivo, we transferred *Gsdmd^−/−^* or *Gsdmd* ^+/+^ neutrophils from CD45.2^+^ donor mice into CD45.1^+^ recipient mice that had been depleted of peripheral neutrophils by Ly6G mAb, transfer efficiency was confirmed by flow cytometry analysis (Figure 1I,J). WT CD45.1^+^ and WT CD45.2^+^ mice exhibited a comparable extent of NETosis activation and tissue damage when undergoing left anterior descending (LAD) ligation (Figure S6). As expected, recipients adopted with *Gsdmd^−/−^* neutrophils (*Gsdmd^−/−^‐*WT) showed less NETs formation, serum LDH, cTnI, CK‐MB, and cardiac IL‐6, IL‐1β, and HMGB1 levels compared to those adopted with *Gsdmd^+/+^* neutrophils (WT‐WT) (Figure 1K–Q). In consist, *Gsdmd^−/−^‐WT* mice were insensitivity to MI/R induced myocardial injury (Figure 1R,S). Taken together, our data demonstrate depletion of neutrophil GSDMD is sufficient to reduce NETs formation and alleviate MI/R injury.

**FIGURE 2:**
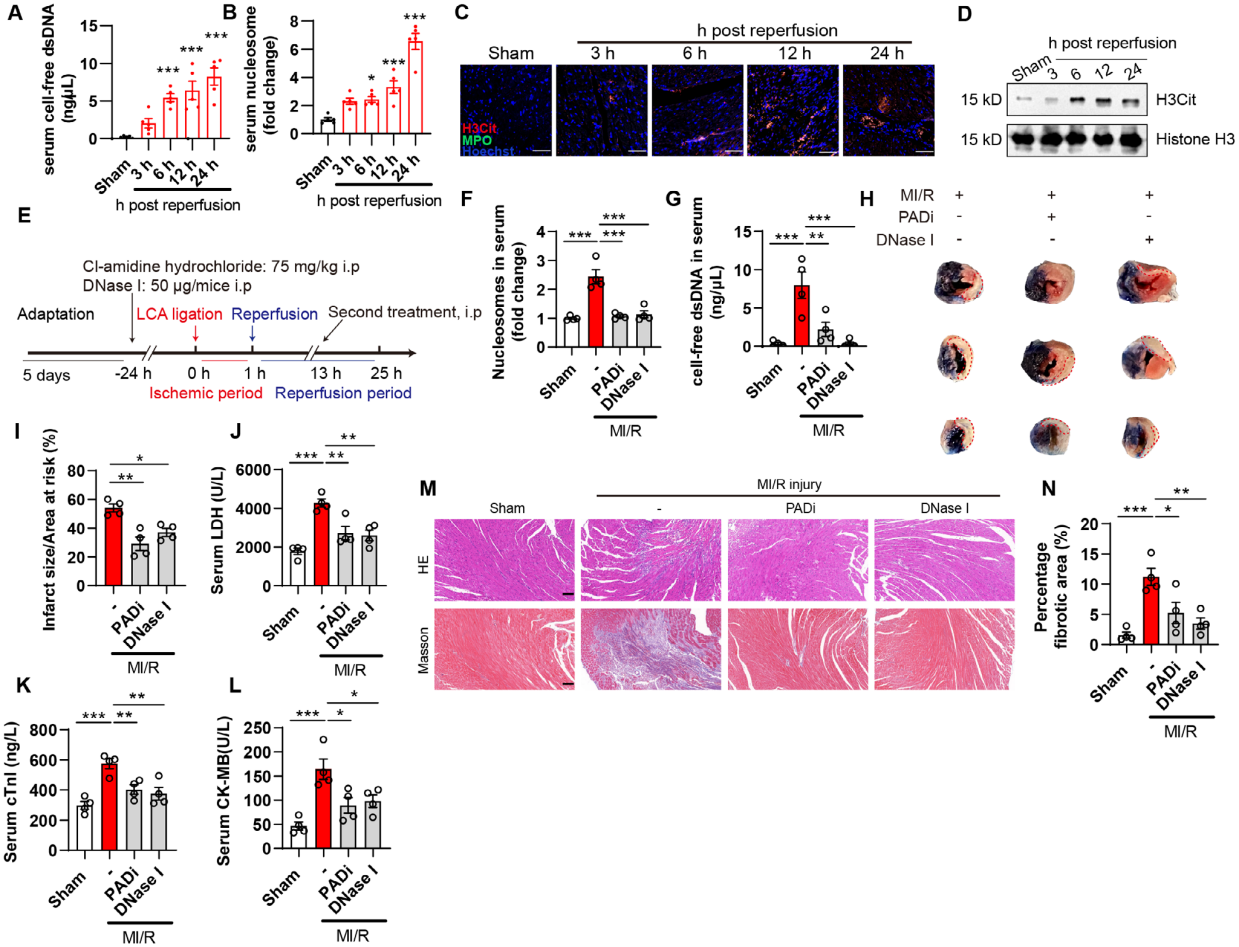
Pharmacological inhibition of neutrophil extracellular trap formation alleviates MI/R injury. (A,B) Analysis of NETosis markers in mice subjected to MI/R injury. Serum cell‐free dsDNA and MPO‐DNA nucleosome complex were determined (*n* = 5). (C) Representative images of heart section with staining for MPO (green), citrullinated Histone H3 (H3Cit, red), and Hoechst (blue). Scale bar, 50 µm. (D) Expression of H3Cit in cardiac tissues determined by immunoblots. ^*^ *p* < 0.05, ^***^ *p* < 0.001 compared to sham group. (E) Work flow of experiment. (F,G) Serum MPO‐DNA nucleosome complex and cell‐free dsDNA were determined (*n* = 4). (H) Representative TTC/Evans blue images of cardiac tissues. The red dotted line indicates the infarct area. (I) Quantification data of infarct size as shown in (H). (J) Serum LDH analysis. (K) Serum cTnI level. (L) Serum CK‐MB level. (M, N) Representative images of HE staining (upper panel) and Masson's trichrome staining (lower panel) of a heart section. Scale bar, 100 µm. *n* = 4 mice per group. Data are shown as mean±SEM. ^*^ *p* < 0.05, ^**^ < 0.01, ^***^ *p* < 0.001 compared to MI/R group.

Thus, we proposed that the infiltration of neutrophils and thereafter NE and/or caspase‐1 mediated GSDMD signal activation may prerequisite for extensive myocardial injury. Indeed, the formation of NETs was confirmed by assessing serum cfDNA and MPO‐DNA nucleosomes (Figure 2A,B). The occurrence of NETosis was evidenced by analyzing H3Cit, a specific marker of NETosis, in cardiac tissues. Notably, NETosis occurred in cardiac tissues as early as 6 h after reperfusion, in advance of the elevation of inflammatory cytokines and noticeable cell death (Figure 2C,D). We next asked whether neutrophils or NETs could directly cause cardiomyocytes injury. Primary neonatal mouse cardiomyocytes (NCMs) were cocultured with primary neutrophils which isolated from mouse bone marrow. We did not observe noticeable toxicity of neutrophil to cardiomyocyte in the coculture system, as indicated by 7‐AAD^+^CD45^−^ cells, which represented dead cardiomyocyte population, indicating that resting neutrophils were incapable of causing myocardial cell damage (Figure S7A,B). We then primed primary neutrophils with phorbol 12‐myristate 13‐acetate (PMA), from which typical NETs were observed. PMA didn't introduce toxicity to NCMs, while PMA pre‐conditioned neutrophils or isolated NETs could directly cause cardiomyocytes death (Figure S7C–G), suggesting NETs was indispensable for neutrophils‐mediated myocardial cell damage. To further verify the causal link between NETs and MI/R injury in vivo, we pretreated mice with NETs inhibitors DNase I (degradation of extracellular DNA) and Cl‐amidine hydrochloride (Pan protein‐arginine deiminase PAD inhibitor), and NETs significantly vanished by both treatments as indicated by serum cfDNA and nucleosomes (Figure 2E–G). Both DNase I and Cl‐amidine hydrochloride were efficient to alleviate extensive cardiac injury at 24 h after reperfusion, as evidenced by reduced infarct size, serum LDH level, serum cTnI, and CK‐MB levels (Figure 2H–L), along with improved cardiac histology (Figure 2M,N). Collectively, these data demonstrate the occurrence of NETosis during the onset of MI/R injury serves as a causal factor in promoting myocardial cell death and exacerbate infarction.

### 2.3 Hypoxia/Reoxygenation Conditioned Cardiomyocytes‐Derived PAF Instigated Neutrophils Undergoing GSDMD‐Dependent NETs Formation and NETosis

**FIGURE 3:**
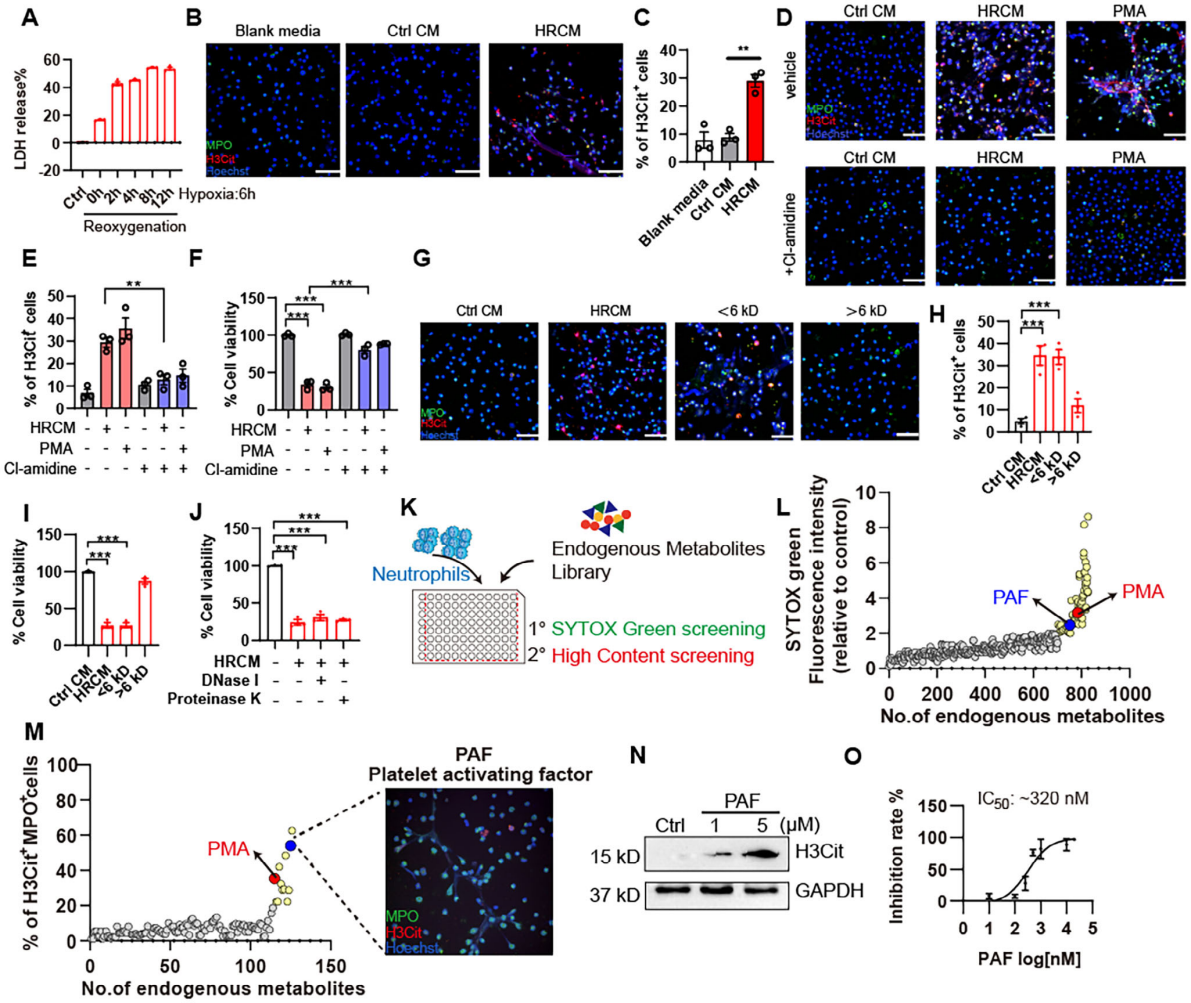
Cardiac cells trigger NETosis during H/R. (A) Primary neonatal cardiomyocytes were subjected to hypoxia for 6 h, followed by reoxygenation for the indicated time points. LDH release were determined (*n* = 3). (B, C) Primary neutrophils were isolated from bone marrow and stimulated with blank media, Ctrl CM, and HRCM for 3 h, representative images (B) with staining for MPO (green), H3Cit (red), and Hoechst (blue), and percentage of H3Cit positive cells (C) were shown (*n* = 3). Scale bar, 50 µm. (D–F) Primary neutrophils were pretreated with 50 µm PAD inhibitor Cl‐amidine for 1 h, followed by treatment of Ctrl CM or HRCM for another 3 h. Representative images with staining for MPO (green), H3Cit (red) and Hoechst (blue), percentage of H3Cit positive cells, and cell viability determined by ATP assay were shown (*n* = 3). Scale bar, 50 µm. ^**^ *p* < 0.01, ^***^ *p* < 0.001 compared to HRCM group. (G–I) Primary neutrophils were treated with Ctrl CM, HRCM, MW < 6 kD fraction or > 6 kD fraction for 3 h. Representative images with staining for MPO (green), H3Cit (red) and Hoechst (blue), percentage of H3Cit positive cells, and cell viability determined by ATP assay were shown (*n* = 3). Scale bar, 50 µm. (J) HRCM were treated with either DNase I or Proteinase K, cell viability was determined by ATP assay (*n* = 3). ^***^ *p* < 0.001 compared to Ctrl CM group. (K) Work flow of endogenous metabolites screening. (L) Fold change of relative fluorescence intensity by each compound detected by SYTOX green, assayed at 40 µm. Cutoff was 2‐fold increase. Yellow dots represent compounds with fold change > 2 (compared to vehicle control) and determined as candidate NETosis triggers. Red dot represents PMA. Blue dot represents PAF. (M) Candidate metabolites were subjected into high content imaging. Percentage of H3Cit and MPO double positive cells were shown. Each compound was assayed at 40 µm. Yellow dots represent positive hits. Red dot represents PMA. Blue dot represents PAF. Right panel, representative image of PAF‐induced NETosis using high content imaging with staining for MPO (green), H3Cit (red), and Hoechst (blue). (N) H3Cit level determined by western blots. (O) IC50 of PAF (*n* = 3). Data are shown as mean±SEM. ^**^ *p* < 0.01, ^***^ *p* < 0.001.

Since NETs were detrimental to cardiac tissue in MI/R injury, we intended to elucidate how NETs were formed during MI/R. NETs formation and NETosis are activated by exogenous pathogen‐associated molecular patterns (PAMPs) and/or endogenous damage‐associated molecular patterns (DAMPs) under infectious conditions, such as TNF‐α, IL‐8, LPS, GM‐CSF, and fMLP [9]. However, NETosis seems to happen in an earlier fashion than inflammatory cytokine secretion in our murine MI/R model; how DAMPs induce NETs and NETosis to cause myocardial injury remains unclear. To ascertain the exact factors that instigate NETosis during MI/R injury, we first assumed whether DAMPs released from the stressed cardiomyocytes would induce NETosis using in vitro hypoxia/reoxygenation (H/R) model. NCMs were subjected into glucose and oxygen deprivation for 6 h, followed by reoxygenation for 2–12 h. H/R caused cell injury time dependently, as determined by LDH release assay (Figure 3A). We collected the conditional medium of NCMs after H/R for 12 h (here we termed HRCM), the conditional medium of NCMs without H/R (termed Ctrl CM), and neutrophil culture medium (blank media), and then primary mouse neutrophils were stimulated with blank media, Ctrl CM or HRCM for 3 h, PMA treatment was used as a positive control to induce NETosis. HRCM induced intensive neutrophil death with extrusion of nuclear component as indicated by H3Cit staining, and forming a web‐like structure similar to PMA induced classical NETs (Figure 3B,C). Notably, conditional medium from normal NCMs which did not undergo H/R process was insufficient to induce NETosis and HRCM‐induced NETs formation can be significantly blocked by PAD inhibitor Cl‐amidine, suggesting HRCM‐induced NETosis was PAD4‐dependent (Figure 3D–F). To probed which component in HRCM is responsible for the induction of NETosis, HRCM was roughly separated into a small molecule fraction (with molecular weight < 6 kD) and a macromolecule fraction (with molecular weight > 6 kD) using ultrafiltration (Figure S8A). The resolution of separation was validated by testing the concentration of lactic acid and LDH (Figure S8B,C). Then, we used the different fractions of HRCM to stimulate primary mouse neutrophils for 3 h, interestingly, only the small molecule fraction induced NETosis, to a comparable extent of HRCM treatment, while the macromolecule fraction was inactive in inducing NETosis (Figure 3G–I). Further, protein and nucleic acid components were degraded from HRCM with protease K and DNase I, separately, both protease K and DNase I conditioned HRCM can induce NETosis in a comparable extent to HRCM (Figure 3J). These results suggested that damaged cardiomyocytes were capable of priming neutrophils to undergo NETosis during H/R, and that secreted metabolic factors may be responsible for the activation of NETosis by cardiomyocytes.

To explore the possible metabolic triggers of NETosis, including those with low concentration under physiological or pathological conditions which could barely be detected by metabolomics analysis, we screened an endogenous metabolites library (containing a total of 829 compounds) to hunt for active compounds in initiating NETosis by Sytox green assay using high throughput screening. Candidate metabolites with cytotoxicity were further screened by high content imaging of H3Cit and MPO double staining to rule out other forms of death such as necrosis. We finally obtained seven metabolites with strong capability to induce NETosis (Figure 3K–M; Tables S2 and S3). Platelet activating factor (PAF), which is an inflammatory mediator released during MI/R in the clinic and has been evidenced to be correlated significantly with risk factors of cardiovascular diseases [15], was among the most effective compounds in activating NETosis with an IC_50_ of 320 nm (Figure 3N,O).

**FIGURE 4:**
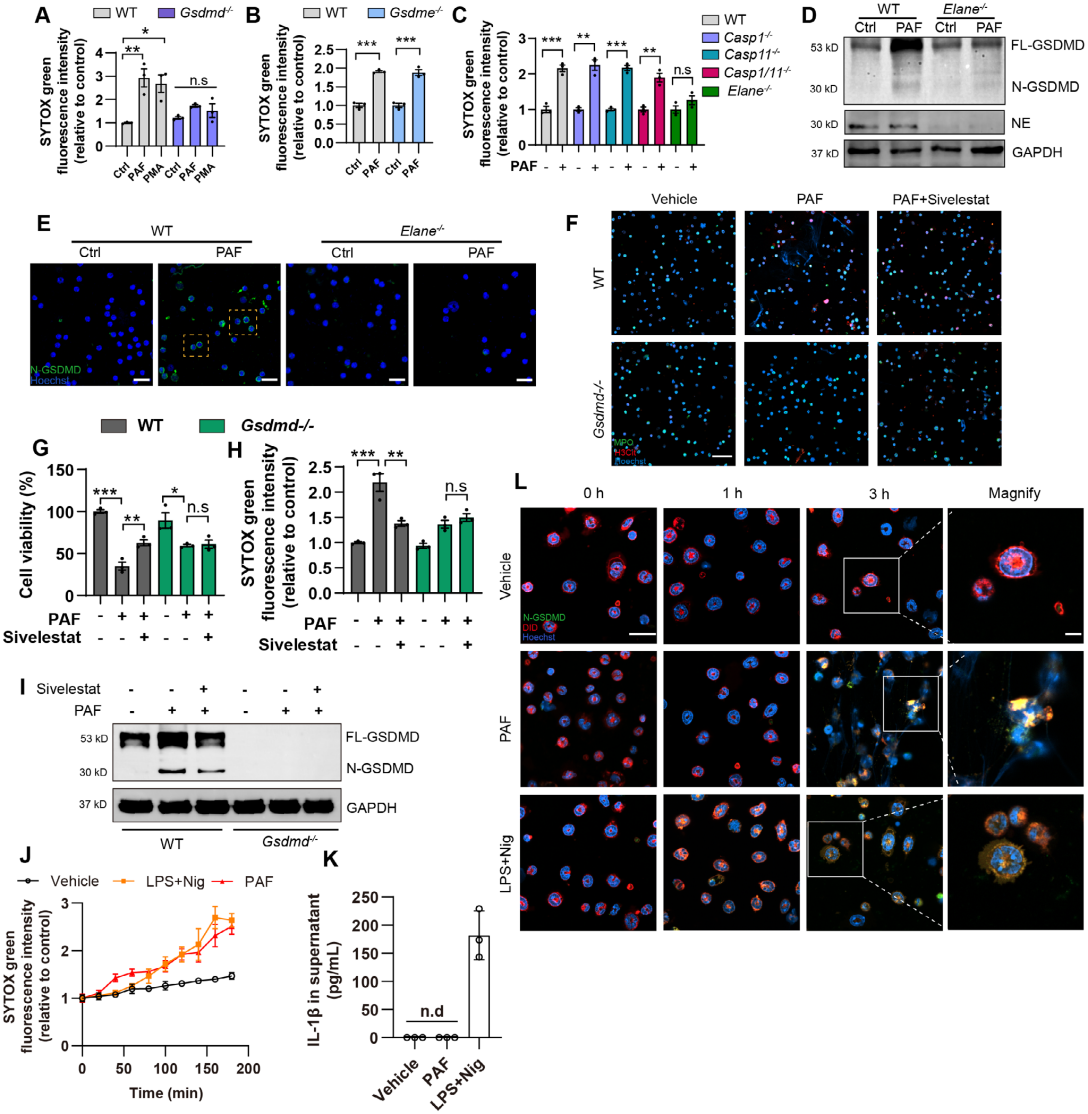
PAF‐induced NETosis is GSDMD‐dependent and independent of caspases. (A) WT and *Gsdmd* ^−/−^ neutrophils were isolated and treated with 100 nm PMA or 5 µm PAF for 3 h. Sytox green‐based NETosis assay were shown (*n* = 3). ^*^ *p* < 0.05, ^**^ *p* < 0.01, n.s., not significant compared to ctrl group. (B) WT and *Gsdme* ^−/−^ neutrophils were isolated and treated with 5 µm PAF for 3 h. Sytox green‐based NETosis assay were shown (*n* = 3). ^***^ *p* < 0.001 compared to ctrl group. (C) WT, *Caspase1* ^−/−^, *Caspase11* ^−/−^, *Caspase1/11* ^−/−^ and *Elane* ^−/−^ neutrophils were treated with 5 µm PAF for 3 h. Sytox green‐based NETosis assay were shown. (D) Primary neutrophils were isolated from WT or *Elane* ^−/−^ mice and treated with 5 µm PAF for 6 h. Maturation of GSDMD was determined by western blot. (E) WT and *Elane* ^−/−^ neutrophils were treated with 5 µm PAF for 1 h. Representative images with staining for N‐GSDMD (green) and Hoechst (blue) were shown. Scale bar, 20 µm. (F) WT and *Gsdmd* ^−/−^ neutrophils were isolated and pre‐treated with 10 µm sivelestat for 1 h, followed by 5 µm PAF treatment for 3 h. Representative images with staining for MPO (green), H3Cit (red), and Hoechst (blue) were shown. Scale bar, 20 µm. (G,H) WT and *Gsdmd* ^−/−^ neutrophils were pretreated with 10 µm sivelestat for 1 h, followed by induction of NETosis using 5 µm PAF for 3 h. ATP‐based cell viability and sytox green uptake‐based NETosis were determined (*n* = 3). (I) Western blot analysis of GSDMD. (J) Primary neutrophils were either treated with 5 µm PAF or primed with 100 ng/mL LPS followed by 10 µm Nigericin treatment. Sytox green‐based cell death were determined every 20 min. (K) IL‐1β in supernatant. (L) Representative images with staining for N‐GSDMD (green), DiD (cell membrane, red), and Hoechst (blue) were shown. Scale bar, 20 µm. Data are shown as mean±SEM, ^**^ *p* < 0.01, ^***^ *p* < 0.001, n.s., not significant compared to each ctrl group.

Previous studies have demonstrated PAF triggers intracellular cascades either in a PAF receptor (PAFR)‐dependent or PAFR‐independent manner [16, 17, 18]. Although PAF has previously reported as an NETosis initiator, the underlying mechanism remains unclear [19, 20]. As MI/R mice exhibited a marked activation of NE, caspase‐1, and GSDMD at the early phase, we asked whether PAF‐activated necroinflammation in neutrophils is associated with NE‐GSDMD or caspase‐1‐GSDMD signaling. *Gsdmd* ^−/−^neutrophils derived from *Gsdmd* ^−/−^ mice, but not *Gsdme^−/−^* neutrophils were resistant to PAF‐mediated NETosis, confirming that PAF‐induced NETosis requires GSDMD (Figure 4A,B). We thus tended to seek out how GSDMD is activated, neutrophils from WT, *Caspase1* ^−/−^, *Caspase11* ^−/−^ *Caspase1/11* ^−/−^, and *Elane* ^−/−^ mice were treated with PAF; NETosis were analyzed by Sytox green assay. Surprisingly, only *Elane* ^−/−^ neutrophils were resistant to PAF‐mediated NETosis (Figure 4C). *Elane* ^−/−^ neutrophils also featured with reduced cleavage and membrane localization of GSDMD upon PAF treatment, suggesting that PAF causes NE‐dependent cleavage of GSDMD in neutrophils (Figure 4D,E). Moreover, we found pharmacological inhibition of NE using sivelestat significantly reduced PAF‐induced NETs formation and GSDMD cleavage in WT neutrophils, while it did not show further inhibition of NETs in *Gsdmd* ^−/−^ neutrophils (Figure 4F–I). It has been disclosed that N‐terminal fragment of GSDMD traffics to neutrophil organelles and facilitates IL‐1β release independently of plasma membrane pores in response to LPS and nigericin treatment [21]. Here, we observed both PAF and nigericin triggered neutrophil death as evidenced by Sytox green assay, however, in comparison to classical inflammasome‐mediated GSDMD processing, PAF did not activate inflammatory caspases and IL‐1β release (Figure 4J,K). Moreover, we found PAF resulted in web‐like structure formation and cell membrane rupture, along with N‐GSDMD colocalization with NETs, while nigericin treatment did not induce typical NETs formation, and both cytosolic and membrane colocalized N‐GSDMD were observed (Figure 4L). Taken together, our findings reveal that NE‐dependent GSDMD cleavage and NETs formation is involved in PAF‐mediated NETosis.

### 2.4 Inhibition of PAF‐GSDMD‐NETosis Signal Axis Alleviates Neutrophils‐Mediated Myocardial Injury in MI/R Mice

PAF is an inflammatory mediator released during MI/R in the clinic and has been evidenced to be correlated significantly with risk factors of cardiovascular diseases [15]. We thus extended to investigate how PAF was accumulated in the myocardium in the process of MI/R. PAF was significantly elevated in cardiac tissue of mice at an early stage of 3 h after reoxygenation, and was also elevated in HRCM from NCMs as soon as 6 h after reperfusion, demonstrating cardiomyocytes could rapidly release PAF upon reperfusion (Figure S9A–C). Notably, when NCMs underwent hypoxia only, we did not observe excessive PAF release or cell death. In myocardial infarction mice, we also did not detect elevated PAF in cardiac tissues, suggesting PAF release requires reperfusion process (Figure S9D–F). PAF can be produced from two synthetic pathways in many cell types, therein the remodeling pathway was mediated by PLA2 and LPCAT2, while *de novo* pathway requires CHPT1 enzyme. Reverse conversion of PAF and lysoPAF by PAF‐acetyltransferase and PAF‐acetylhydrolase also contributes to regulating PAF homeostasis (Figure S9G) [22]. We next evaluated the myocardial expression levels of key enzymes in PAF synthesis and metabolism pathways, and found that *Pla2g6*, a key enzyme involved in remodeling pathway, was significantly upregulated both in vivo in MI/R mice and in vitro in NCMs under H/R condition (Figure S9H,I). Selective PLA2G6 inhibitor S‐Bromoenol lactone (S‐BEL), but not PLA2G7 inhibitor darapladib or PLA2G4A inhibitor pyrrophenone, was capable of inhibiting PAF production in H/R conditioned NCMs, suggesting that PAF production in injured cardiomyocytes relies on PLA2G6 (Figure S9J). Treatment of S‐BEL also retarded HRCM induction of NETosis (Figure S9K,L).

**FIGURE 5:**
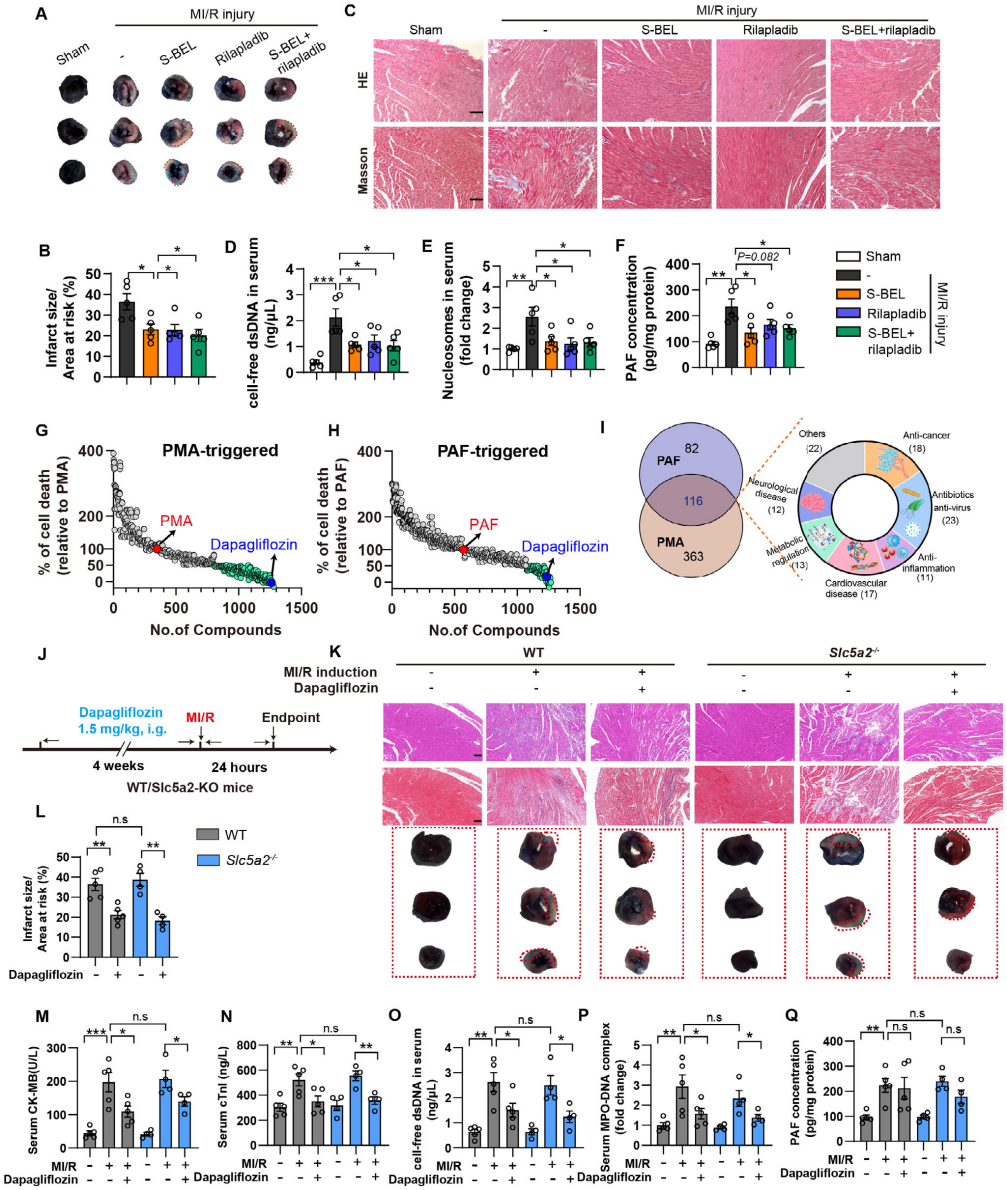
Pharmacological manipulation of PLA2G6‐PAF‐NETosis axis alleviates MI/R injury. Mice were intraperitoneally pretreated with 10 mg/kg S‐BEL, 10 mg/kg rilapladib and combination of S‐BEL and rilapladib 12 h before LAD ligation, respectively, followed by reperfusion for 24 h. (*n* = 5). (A) Representative images of TTC/Evans Blue staining. Red dotted lines indicate the infarct area. (B) Percentage of infarct size within the area at risk. (C) Representative image of HE staining (upper panel) and Masson's trichrome staining (lower panel) of heart section at different time points. Scale bar, 100 µm. (D–F) serum dsDNA, MPO‐DNA nucleosome complex and cardiac PAF concentration were determined. ^*^ *p* < 0.05, ^**^ *p* < 0.01, ^***^ *p* < 0.001 compared to MI/R group. (G, H) High throughput screening of FDA‐approved compound library basing PMA‐ and PAF‐induced NETosis models. Relative cell death ratio was determined by using SYTOX green assay. Green dots represent compounds with less than 50% cell death rate, PMA and PAF serves as positive control and is considered as 100% cell death, red dot represents PMA or PAF, blue dot represents dapagliflozin. (I) Venn diagram (left panel) and summary of 116 active compounds which showed inhibition rate over 50% in both PMA‐ and PAF‐induced NETosis. (J) Schematic illustration of experimental design. WT and *Slc5a2* ^−/−^ mice were orally gavaged with 1.5 mg/kg dapagliflozin for 4 weeks, followed by MI/R induction. (K) Representative images of HE staining (upper panel, scale bar, 100 µm), Masson's trichrome staining (middle panel, scale bar, 100 µm), and TTC/Evans Blue staining (lower panel) were shown. Red dotted lines indicate the infarct area. (L) Measurement of infarct size (*n* = 4‐5). (M) Serum CK‐MB level. (N) Serum cTnI level. (O, P) Serum cell‐free dsDNA and MPO‐DNA nucleosome complex were determined. (Q) Cardiac concentration of PAF determined by ELISA (*n* = 5). Data are shown as mean±SEM. ^*^ *p* < 0.05, ^**^ *p* < 0.01, ^***^ *p* < 0.001. n.s., not significant.

To validate if PAF metabolism dysregulation is pivotal for MI/R injury, we treated mice with PLA2G6 inhibitor S‐BEL, PAFR inhibitor rilapladib, and the combination of S‐BEL and rilapladib before MI/R surgery. We found that either inhibition of PLA2G6 or PAFR was sufficient to alleviate MI/R injury, as evidenced by reduced infarct size and histological measurements (Figure 5A–C). Notably, S‐BEL reduced PAF concentration in cardiac tissue, and both S‐BEL and rilapladib treatment significantly reduced NETs formation in MI/R mice as evidenced by serum nucleosomes and cell‐free dsDNA concentrations (Figure 5D–F). We further assessed if inhibition of PLA2G6‐mediated PAF production by S‐BEL would further protect *Gsdmd* ^−/−^ mice form MI/R injury. As expected, PLA2G6 inhibition in WT mice could significantly alleviate cardiac injury, while it did not show further protection in *Gsdmd* ^−/−^ mice, suggesting that PAF aggravating MI/R injury is largely dependent of GSDMD/NETosis pathway (Figure S9M–P). Collectively, these data demonstrate cardiomyocyte‐derived PAF triggered NETosis is a key incentive of MI/R injury, accumulation of PAF in injured myocardial tissue was closely related to the upregulation of PLA2G6.

To further identified potential inhibitor of NETosis, we screened a library of 1499 FDA‐approved drugs using PMA‐ and PAF‐triggered NETosis models. Generally, primary mouse neutrophils were isolated and pre‐treated with 20 µm drug compound for 1 h followed by induction of NETosis with 100 nm PMA or 5 µm PAF for 3 h, then 100 nm Sytox green DNA probe was added and incubated at room temperature for 15 min for detection of cell death. Here, we identified 116 drugs with strong potential in inhibiting both PMA‐ and PAF‐triggered NETosis (Figure 5G–I; Table S4). Among these drugs, we found SGLT2 inhibitor dapagliflozin exhibited strong inhibition efficacy (approximately 80%). Dapagliflozin inhibited NET formation and NETosis, along with less N‐GSDMD production under PMA and PAF treatments (Figure S10A–C). Notably, the inhibitory effect of dapagliflozin on NETosis was independent of SGLT2, as dapagliflozin showed an equivalent inhibitory effect on NETosis in *WT* and *Slc5a2* ^−/−^ neutrophils (Figure S10D–F). We further asked if other SGLT2 inhibitors including empagliflozin, canagliflozin, and ertugliflozin could be also effective in inhibiting NETosis, interestingly, all these SGLT2 inhibitors exhibited protective effect on NETosis also in a SGLT2‐independent manner, but to a minor extent in comparison with dapagliflozin (Figure S10G,H). Dapagliflozin has been shown to reduce mortality rates and cardiovascular events among patients with or without type 2 diabetes, while the underlying mechanism remains largely unexplored [23, 24]. We herein hypothesized that dapagliflozin could alleviated MI/R injury by inhibiting NET formation. To test this hypothesis, mice were pretreated with 1.5 mg/kg dapagliflozin for 4‐weeks followed by MI/R induction. As expected, dapagliflozin significantly alleviated cardiac infarct size, fibrosis, and NETosis, without affecting the level of PAF in cardiac tissues, while the cardioprotective effect of dapagliflozin is SGLT2‐independent, as dapagliflozin also exhibited significant cardioprotective effects in *Slc5a2* ^−/−^ mice (Figure 5J–Q). Furthermore, CD45.1 mice were treated with Ly6G neutralizing antibody and transplanted with either CD45.2 WT neutrophils or CD45.2 *Gsdmd* ^−/−^ neutrophils, 50 mg/kg dapagliflozin were administered intraperitoneally 12 h before MI/R induction. In mice transplanted with *Gsdmd* ^−/−^ neutrophils, dapagliflozin was incapable of protecting mice from MI/R injury, indicating that GSDMD‐NETosis signal pathway is indispensable for mediating dapagliflozin cardioprotection activity (Figure S11). Collectively, these results evidenced the casual association between dapagliflozin and GSDMD‐dependent NETosis. In conclusion, our results not only confirm that inhibition of GSDMD‐NETosis pathway in peripheral neutrophils may provide therapeutical benefits during MI/R injury, but also provide new therapeutical drugs for prevention of MI/R injury.

### 2.5 Dapagliflozin Alleviates Cardiac Injury via Engaging LCN2

**FIGURE 6:**
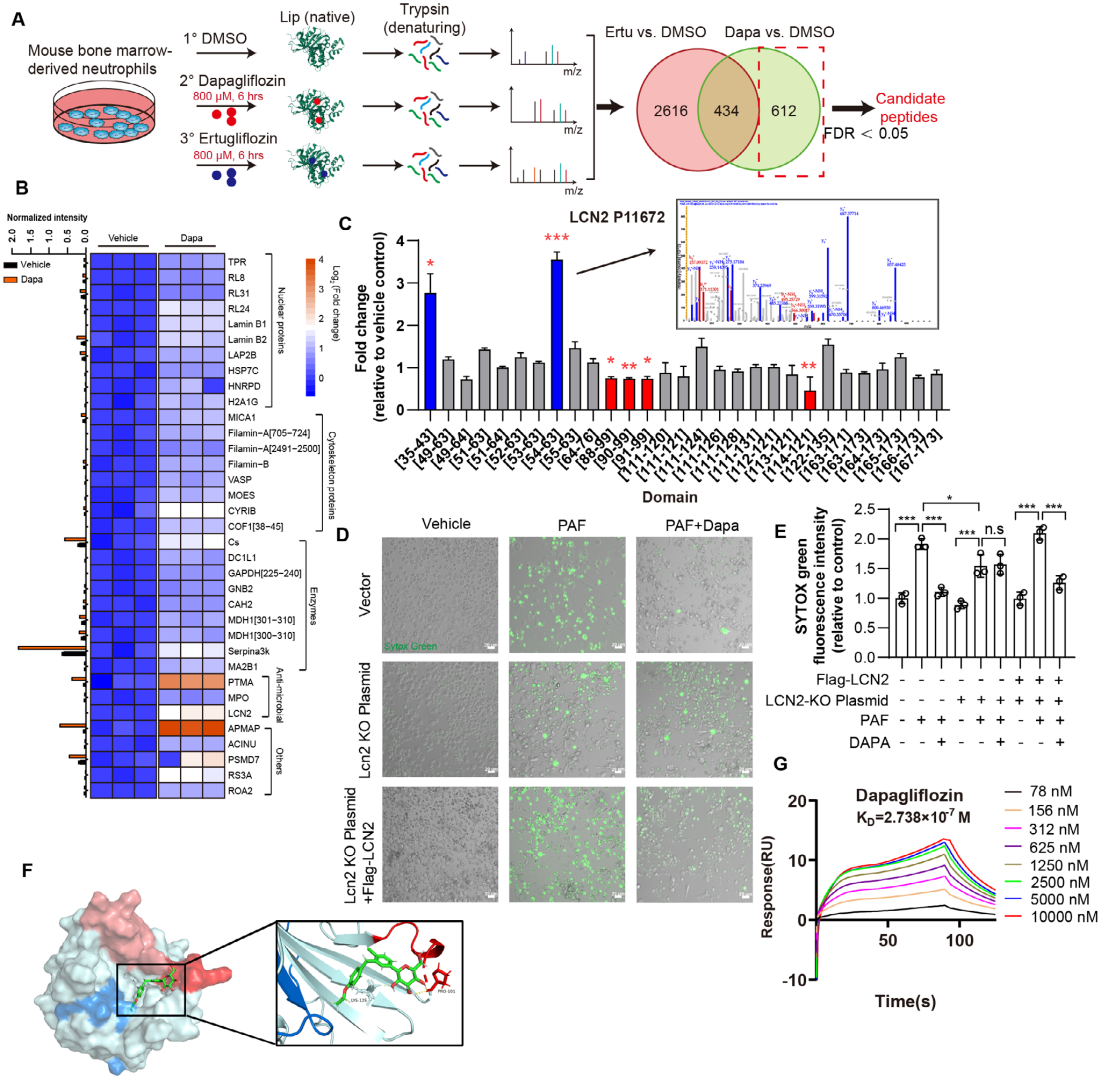
Identification of potential dapagliflozin targets using LiP‐MS. (A) Flow chart depicting the LiP‐MS assay. Primary neutrophils were isolated from mouse bone marrow and treated with DMSO, 800 µm dapagliflozin, and 800 µm ertugliflozin for 6 h, followed by Lip native digestion and analysis by MS. Ertugliflozin serves as a negative control. Differentiated peptides were identified between the Dapa group and Vehicle group and proteins which is differentially expressed between Ertu group and Vehicle group were excluded. The Candidate protein peptides were then selected under the criteria as follows: fold change >1.5 and FDR adjusted p‐value < 0.05. (B) Heatmap of 66 differentially expressed protein peptides between Dapa group and Ctrl group. Red peptides represent FDR < 0.05 in Dapa group while FDR > 0.05 in Ertu group when compared to vehicle ctrl group. (C) Fold change of indicated LCN2 protein domains detected by LiP‐MS. Relative mass spectrometry images obtained from dapagliflozin group were shown. (D) dHL‐60 cells were transfected with NC or NGAL CRISPR/Cas9 KO Plasmid and expressed Flag‐LCN2. Cells were then pre‐treated with 20 µm dapagliflozin for 1 h, followed by induction of NETosis by 20 µm PAF for another 3 h. Relative confocal images were shown. Green, Sytox probe. Scale bar = 20 µm. (E) Sytox green based cell death were determined (*n* = 3). (F) Binding mode of dapagliflozin with LCN2 (PDB:3HWE), data were generated by AutoDock Vina 1.1.2 and further visualized by Pymol 2.5.7. Red‐indicated domains are resistant to enzymatic digestion and blue‐indicated are more enzymatic accessible, as related to data from Lip‐MS in Figure 6C. (G) Binding affinities of dapagliflozin to recombinant LCN2 protein were detected by SPR assay; equilibrium dissociation constant (KD) is indicated. Data are shown as mean±SEM. ^*^ *p* < 0.05, ^**^ *p* < 0.01, ^***^ *p* < 0.001. n.s., not significant.

To decipher the potential therapeutic target of dapagliflozin in inhibiting NETosis and ensuing myocardial infarction, we applied an approach of limited proteolysis‐coupled mass spectrometry (LiP‐MS) to reveal the interactome of dapagliflozin in primary mouse neutrophils, ertugliflozin was parallelly investigated as a negative control to exclude functional unrelated interacting proteins as it is inactive in inhibiting NETosis. Primary mouse neutrophils were treated with dapagliflozin, ertugliflozin, or vehicle control as indicated, cells were collected followed by sequential limited digestion and complete digestion. Finally, label‐free proteomics analysis is applied to identify structure‐dependent proteolytic patterns corresponding to protein targets of dapagliflozin as well as potential binding sites (Figure 6A). 613 kinds of proteins were specifically enriched in dapagliflozin group, and the top fold‐changed proteins were listed out in heatmap, with indication of intensity (Figure 6B; Table S5). To determine the exact target for dapagliflozin binding, we conducted in silico analysis on the seven most potential targets derived from the aforementioned experiments, including SerpinA3k (protein ID: P01011), PTMA (protein ID: P06454), PSMD7 (protein ID: O00231), APMAP (protein ID: Q9HDC9), CO3 (protein ID: P01024), CS (protein ID: O75390) and neutrophil‐derived lipocalin‐2 (LCN2, protein ID: P80188). Specifically, molecular docking in conjunction with structure analysis was conducted to rank the potential targets. Five crystal structures and two AlphaFold2 (AF2) structures [25] were collected for the analysis (see Method section). Since PTMA holds a disordered structure and there is no known active site for drug binding in SerpinA3k, PSMD7, and APMAP, docking‐based analysis could only be conducted on CS (−5.9 kcal/mol), LCN2 (−5.4 kcal/mol), and CO3 (−5.4 kcal/mol) (Figure S12A). Synchronously, we performed an mRNA silencing experiment in differentiated HL‐60 (dHL‐60) cells to screen the functional gene that mediates the inhibitory effect of dapagliflozin on NETosis. Silencing LCN2 attenuated dapagliflozin's protective effect against NETosis, as measured by cytotoxicity assays, this phenotype was rescued by LCN2 overexpression, confirming LCN2 as a functional target of dapagliflozin (Figure 6C–E; Figure S12B,C). Moreover, we conducted a comparative experiment on other three SGLT2i compounds (empagliflozin, canagliflozin, and ertugliflozin) for engaging LCN2, which shows decreased activities compared to dapagliflozin in inhibiting NETosis (Figure S10G,H). Consistently, our in silico analysis captured the experimental trend, with dapagliflozin showing the strongest binding affinity to LCN2 (−5.4 kcal/mol), empagliflozin the second (−5.1 kcal/mol), and canagliflozin (−4.1 kcal/mol) and ertugliflozin (−4.7 kcal/mol) the lasts. A potential reason for the weakened affinity for canagliflozin and ertugliflozin may arise from the lost H‐bonds and/or cation‐π interaction between the ligands and the receptor. As shown in Figure 6F and Figure S12D, four H‐bonds and one or two cation‐π interaction(s) formed in the systems of dapagliflozin and empagliflozin, respectively, whereas, only three H‐bonds and none or one cation‐π interaction were found in the systems of canagliflozin and ertugliflozin, respectively, which may seriously weaken the drugs’ activity to LCN2. Moreover, the specific interaction of dapagliflozin and recombinant human LCN2 was validated by SPR analysis. Our data showed that only dapagliflozin exhibited a potential to interact with LCN2, with a KD value of 2.7 × 10^−7^ m (Figure 6G; Figure S12E–G). In conclusion, our results hint that the cardioprotective effects of dapagliflozin in ameliorating MI/R injury are mediated through a novel SGLT2‐independent pathway. Specifically, dapagliflozin suppresses PAF‐induced NETosis by directly targeting LCN2, thereby alleviating inflammatory damage in myocardial tissue.

**FIGURE 7:**
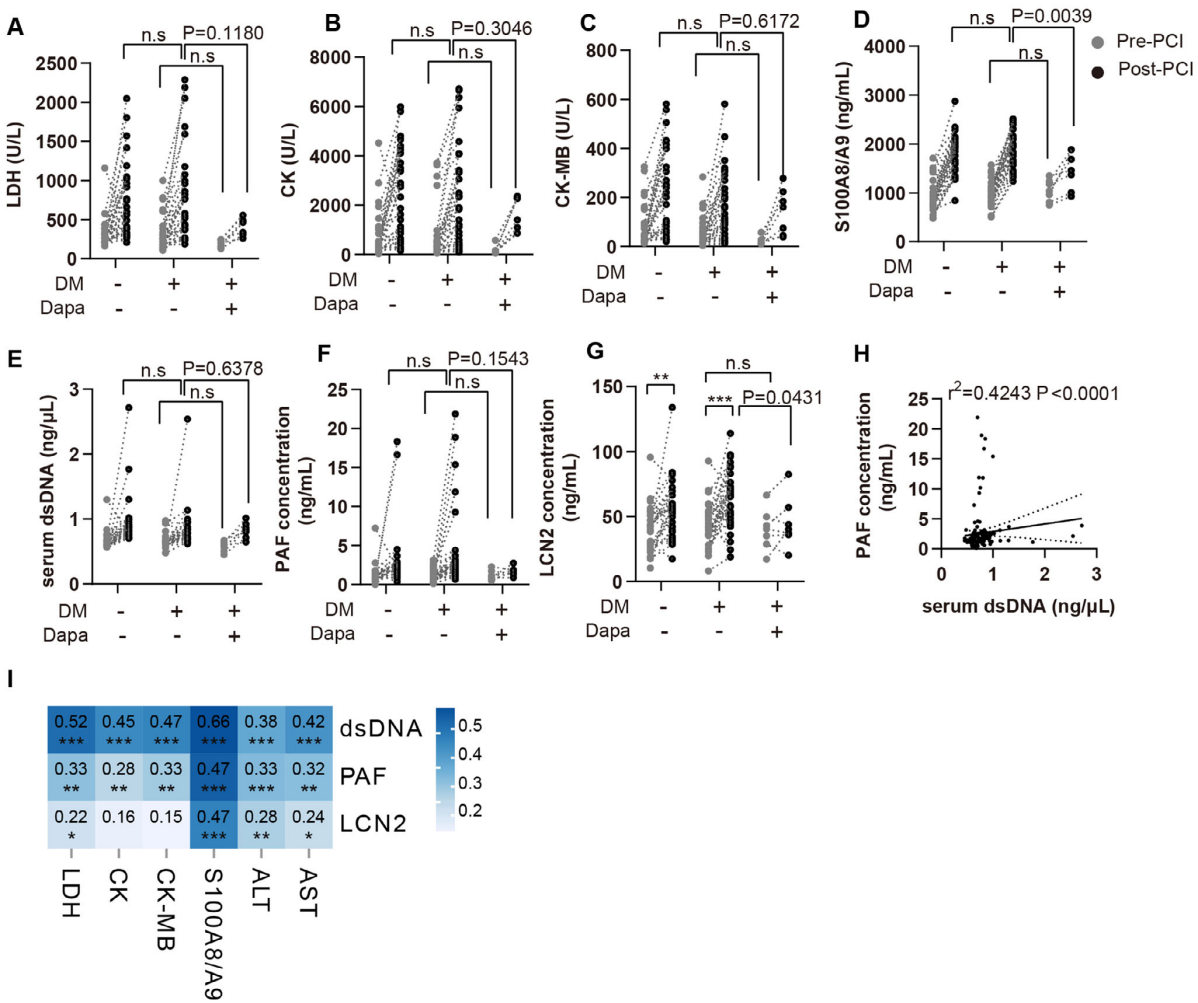
Patients with percutaneous coronary intervention (PCI) exhibited elevated NETs formation and PAF. (A–D) Serum LDH, CK, CK‐MB and S100A8/A9 were determined before and after PCI in patients either with diabetes mellitus (DM) or not (*n* = 33 for patients with DM and do not receive dapagliflozin treatment, *n* = 7 for patients with diabetes mellitus and received long‐term dapagliflozin treatment. *n* = 32 for patients without DM). (E) Serum cell‐free dsDNA was determined before and after PCI. (F) Serum PAF concentration was determined using ELISA before and after PCI. (G) Serum LCN2 concentration was determined using ELISA before and after PCI. (H) Spearman's correlation analysis between serum dsDNA and PAF. (I) Spearman's correlation analysis between each parameter (*n* = 144). ^*^ *p* < 0.05, ^**^ *p* < 0.01, ^***^ *p* < 0.001.

To further translationally valid our findings, a total of 72 AMI patients with PCI surgery were enrolled, among which 40 patients were complicated with type 2 diabetes mellitus (Table S6). Serum NETs and PAF concentration in AMI patients’ peripheral blood before and 6 h after PCI surgery were investigated. The enrolled AMI patients with PCI surgery exhibit high serum levels of LDH, CK, CK‐MB, and S100A8/A9, along with elevated serum cfDNA, LCN2, and PAF concentrations (Figure 7A–G). Notably, there are 7 AMI patients with diabetes having long‐term medication of dapagliflozin, we found that dapagliflozin strikingly downregulated post‐PCI cardiac injury and NETosis indexes, supporting that alleviation effect of dapagliflozin on PCI prognosis is correlated with inhibiting neutrophil NETosis (Figure 7A–G). Serum PAF concentration was positively correlated with the level of cfDNA, suggesting that PAF was associated with NETs formation in clinical settings (Figure 7H). Additionally, serum cfDNA, LCN2, and PAF level were positively correlated with LDH, CK, CK‐MB, and S100A8/A9 (Figure 7I). Collectively, these clinical data further support that NETosis is associated with poor prognosis in AMI patients undergoing PCI surgery, and that long‐term use of dapagliflozin may have therapeutical benefits in patients with high risks of myocardial infarction.

## 3 Discussion

Although the morbidity and mortality from AMI decreased due to the successful use of interventional coronary reperfusion strategies, patients who did not receive treatment timely still have a high mortality rate [26]. The most important factor which contributes to the prognosis is irreversible cardiac tissue injury during the ischemia/reperfusion period [27], a wide range of pathological processes have been proved to contribute to cardiac tissue injury, such as activation of cell death programs including apoptosis, autophagy, and necrosis. However, therapeutic interventions targeting cardiomyocytes death are mostly disappointing when meeting the clinic [28]. Therefore, a better understanding of molecular mechanisms involved in the early onset of ischemia/reperfusion injury is needed to provide new insights into tissue damages. It is noteworthy that sterile inflammation occurred during ischemia and reperfusion, host‐derived molecules would act as damage‐associated molecular patterns (DAMPs) to trigger the innate immune response and initiate a sterile inflammation in the myocardium, such as mitochondrial DNA, cardiolipin, and ATP which have been proved to be related to activate immune responses without pathogen invasion [29, 30]. Thus, identifying the key DAMPs and inhibiting their early inflammatory signaling pathway may provide new insights for reducing infarct size and the risk of developing post‐infarct heart failure. Here, we identified massive infiltration of neutrophils in the heart of mice suffered from MI/R injury. Interestingly, it has been reported that neutrophils are required for the late‐phase tissue repair through regulating the polarization of macrophages [31], our data demonstrated that in the early stage of MI/R injury, ablation of neutrophils using neutralizing antibody showed therapeutic benefits in mice, suggesting the multifaceted role of neutrophils during MI/R injury. In this study, we find the formation of NETs is decisive for sterile inflammation and tissue damage during MI/R injury, and identified that PAF was a key DAMP in initiating NETs formation and NETosis during pathological process of MI/R. Inhibiting NETs formation using a pharmacological approach by SGLT2 inhibitor dapagliflozin could alleviate sterile inflammation and subsequent myocardial injury in MI/R mice. Specifically, dapagliflozin inhibited NETosis by targeting LCN2 rather than SGLT2, suggesting that inhibition of LCN2 may provide therapeutic approaches for alleviating MI/R prognosis.

It has been widely noted that myocardial infarction triggers an intense inflammatory reaction characterized by infiltration of the infarcted heart with leukocytes, which can adhere to viable cardiomyocytes and may exert cytotoxic effects extending ischemic injury [32]. Targeting inhibition of leukocyte activation, adhesion, and extravasation were successful in attenuating MI/R injury in experimental studies, but translation of leukocyte‐focused treatment into therapy for human populations with myocardial infarction was unsuccessful and several anti‐inflammatory approaches failed to reduce infarct size in clinical investigations [33, 34]. One of the potential reason is that inflammation is reduced by anti‐inflammatory cytokines in the resolution phase, but a low‐grade chronic inflammation continues throughout the post‐phase of MI/R [35]. Thus, a better understanding of the temporal mechanism of how leukocyte activation and exerting cytotoxic effects on cardiomyocytes is urgent for drug development. Increasing evidences indicate that NETs formation and NETosis is critical for neutrophils to participate sterile inflammation and pathogenesis of organ injury, including atherosclerosis, ischemic injury of the kidney and lung [36, 37, 38]. Of note, various metabolic factors are found to be able to cause NETosis, for instance, cholesterol causes NETosis and primes macrophages for production of inflammatory cytokines to amplify immune responses during atherosclerosis [39], high glucose level in diabetic mice has been proved to be responsible for the formation of NETs and impairs wound healing [40], and arachidonic acid metabolism pathway is found to hyperactivated to generate excessive metabolite 12‐hydroxyeicosatetraenoic acid to promote pro‐inflammatory signaling and apoptosis [41]. Till now, which factor is critical contributing to NETosis during MI/R remains elusive. Here, we screened an endogenous metabolites library and identified PAF as a strong NETosis activator which is significantly upregulated during the early onset of MI/R. Previous studies found the release of TNF‐α, HMGB1, and ROS from dead cells are the main causes of NETosis during MI/R injury. However, we observed NETs formation earlier than cytokines burst, PAF is upregulated as early as 3 h after reperfusion, which might explain the early occurrence of NETosis.

PAF is considered as an important inflammation regulator and have been proved to cause NETosis. It has been reported that low concentrations of PAF exert cardioprotective effects, while high concentrations of PAF could worsen post‐ischemic myocardial injury [42]. Here, we identified that the accumulation of PAF is possibly due to the altered PAF remodeling pathway, upregulation of PLA2G6, the key enzyme in PAF metabolism during MI/R injury is responsible for PAF over production upon perfusion or reoxygenation. Although PAF is considered to cause NETosis, the exact mechanism of how PAF initiates NETosis remains unknown. Recent studies indicated that GSDMD is involved in PMA‐induced NETs formation [43, 44]. The maturation of GSDMD to generate N‐terminal fragment of GSDMD is responsible for DNA extrusion upon NETosis. To interrogate whether GSDMD is also required during PAF‐induced NETosis, we assessed the capacity of NETs formation in *Gsdmd* ^−/−^ neutrophils. PAF treatment results the cleavage of GSDMD, *Gsdmd* ^−/−^ neutrophils were resistant to PAF. Interestingly, PAF activates GSDMD rely on NE, rather not Caspase‐1 or Caspase‐11, as *Elane* ^−/−^ neutrophils results less GSDMD cleavage upon PAF treatment. Further research is warranted to study how PAF release NE from azurophilic granule to activate GSDMD. *Gsdmd* ^−/−^ mice are protected from MI/R injury, confirming that inhibition of NETosis is promising for the early treatment of MI/R injury. Overall, our findings demonstrated that NETs formation during reperfusion period could exacerbates inflammatory responses and tissue damage. Altered metabolism of PAF resulted elevated PAF concentrations in the ischemic heart which was responsible for early NETosis. PAF‐mediated NETosis is GSDMD‐dependent which require NE activity.

Given that pharmacological strategy of both PAF and GSDMD/NETosis signaling nodes are unavailable in the clinic, we screened by an FDA‐approved drug library to seek out a potential therapeutic approach. Interestingly, we found a SGLT2 inhibitor, dapagliflozin, were capable of inhibiting PAF‐NETosis and subsequently MI/R injury, in a SGLT2‐independent manner. In consist with previous large‐scale trails which assessed cardiovascular outcomes with the SGLT2 inhibitors including dapagliflozin, ertugliflozin, empagliflozin, and canagliflozin, in T2DM patients who were at high risk for cardiovascular events [45, 46, 47], SGLT2 inhibition, especially medication of dapagliflozin, was correlated with a lower rate of hospitalization for heart failure. Recent studies also indicated that dapagliflozin could lower the risk of worsening heart failure or death from cardiovascular causes regardless of the presence or absence of diabetes, possibly through glucose‐independent mechanisms [23, 48, 49]. Here we identified by a Lip‐MS method that dapagliflozin is a potent NETosis inhibitor which can efficiently block NETs formation by direct target LCN2, which has also been recognized as a risk factor of cardiovascular diseases [50]. Our results may partially explain the intrinsic mechanism of the inflammatory modularity and the protective effects of dapagliflozin on cardiovascular diseases. However, there is currently no direct experimental evidence demonstrating how LCN2 regulates PAF‐induced NETosis. Previous studies have demonstrated that PAF‐triggered NETosis involves PAFR activation and NADPH oxidase (NOX)‐dependent ROS generation, while LCN2 has been shown to amplify neutrophil ROS production and is vital for NETosis [51, 52]. Although in bacteria‐induced NETosis, genetic deletion of *Lcn2* gene did not affect NETs formation but reduced the bactericidal effect of NETs [53], here we evidenced both LCN2‐silenced HL‐60 cells and primary neutrophils from *Lcn2* ^−/−^ mice were insensitive to PAF induced NETosis, future studies are needed to clearly demonstrate the exact mechanisms of how LCN2 regulates PAF‐induced NETosis. Overall, this study provides new insight into metabolic factors in regulating host immune responses through NETosis, and also proposes a potential strategy to inhibit NETosis by LCN2 inhibitors during myocardial MI/R injury.

Preclinical studies demonstrate that interventions using DNase I or Cl‐amidine hydrochloride, attenuate microvascular obstruction and preserve cardiac function in murine models, however, targeting neutrophil‐mediated inflammation for ischemic tissue injury remains challenging due to the pleiotropic roles of neutrophils in infection control and tissue toxicity. Despite LCN2 explained the essence of dapagliflozin cardioprotective efficacy, our proposed MI/R intervention by LCN2i may also face similar issues when turn to therapy development, further exploration is required to precisely modulate NETosis to balance anti‐infection and inflammatory injury pathways. Emerging evidence also implicates neutrophil heterogeneity in MI/R progression, thus, future research of LCN2i drug development necessitate context‐specific therapeutic strategies.

## 4 Experimental Section

### 4.1 Reagents and Antibodies

Endogenous Metabolite Compound Library (HY‐L030), FDA‐Approved Drug Library (HY‐L022), Cl‐amidine hydrochloride (HY‐100574A), VX‐765 (HY‐13205), Z‐DEVD‐FMK (HY‐12466), Necrostatin‐1 (HY‐15760), Sivelestat (HY‐17443), C16 PAF (HY‐108635), LDC7559 (HY‐111674), Rilapladib (HY‐102004), Dapagliflozin (HY‐10450), Empagliflozin (HY‐15409), Canagliflozin (HY‐10451), Ertugliflozin (HY‐15461) and Darapladib (HY‐10521) were obtained from MedChemExpress (Shanghai, China). DNase I (D4513), PMA (P1585) and All‑trans retinoic acid (ATRA) (R2625‐50MG) were obtained from Sigma‐Aldrich (Saint Louis, MO, USA). (S)‐Bromoenol Lactone (GC40679) was obtained from GLPBIO (Montclair, NJ, USA). Pyrrophenone (13294) was obtained from Cayman Chemical (Ann Arbor, MI, USA). Neutrophil elastase antibody (A8953) was obtained from Abclonal (Wuhan, China). Anti‐Histone H3 (citrulline R2+R8+R17) antibody (ab5103), anti‐Histone H3 antibody (ab1791), and anti‐GSDMD antibody (ab209845) were obtained from Abcam (Cambridge, UK). Anti‐MPO antibody (AF3667) were obtained from NOVUS Biologicals (Centennial, CO, USA). Cleaved Gasdermin D (#10137) was obtained from Cell Signaling Technology (Boston, MA, USA).

For antibodies used in flow cytometry, Brilliant Violet 605 anti‐mouse/human CD11b Antibody (101257, clone M1/70), Pacific Blue anti‐mouse CD45 Antibody (103126, clone 30‐F11), PerCP/Cyanine5.5 anti‐mouse Ly‐6G Antibody (127616, clone 1A8), Brilliant Violet 510 anti‐mouse CD103 Antibody (121423, clone 2E7), APC anti‐mouse Ly‐6C Antibody (128016, clone HK1.4), PE anti‐mouse F4/80 Antibody (123110, clone BM8), Alexa Fluor 700 anti‐mouse CD11c Antibody (117320, clone N418), 7‐AAD Viability staining solution (420404) and Zombie NIR fixable viability dye (423105) were purchased from Biolegend (San Diego, CA, USA).

For antibodies used in neutrophil depletion, InVivoMAb anti‐mouse Ly6G (BE0075‐1, clone 1A8) and InVivoMAb rat IgG2α isotype control, anti‐trinitrophenol (BE0089, clone 2A3) were obtained from Bio X Cell (Lebanon, NH, USA).

### 4.2 Participants

72 AMI patients with PCI surgery from the Clinical Biological Sample Library in Nanjing Drum Tower Hospital, the Affiliated Hospital of Nanjing University Medical School were enrolled in the present study. Blood samples were collected before and 6 h post PCI surgery. All human studies were approved by the Ethics Committee of the Affiliated Drum Tower Hospital of the Medical School of Nanjing University (Permit Number: 2019–190‐01; 2021‐531‐02). All subjects gave informed consent. The main characteristics of patients are shown in Table S6

### 4.3 Animals

C57BL/6 J male mice (6–8 weeks old) were obtained from GemPharmatech Co., Ltd (Nanjing, China) and SLAC Laboratory Animal (Shanghai, China). *Gsdmd* knockout (*Gsdmd^−/−^*), *Gsdme* knockout (*Gsdme^−/−^*), *Caspase1* knockout (*Caspase1^−/−^*), *Caspase11* knockout (*Caspase11^−/−^*) and *Caspase1/11* knockout (*Caspase1/11^−/−^*) mice were all C57BL/6 background and kindly provided by Dr. Feng Shao at National Institute of Biological Sciences (Beijing, China). *Elane* knockout (*Elane* ^−/−^) mice were kindly provided by Dr. Aimin Xu at The University of Hong Kong (Hongkong, China). *Slc5a2* knockout (*Slc5a2* ^−/−^) mice were obtained from Jiangsu Aniphebio company (Nanjing, China). *Lcn2* knockout (*Lcn2* ^−/−^) mice were obtained from Cyagen (Suzhou, China). CD45.1 and CD45.2 C57BL/6 JGpt male mice (6–8 weeks old) were obtained from GemPharmatech Co., Ltd. All animal procedures were approved by the Institutional Animal Care and Use Committee of China Pharmaceutical University (Approval number: 2021‐05‐009).

### 4.4 Differentiation of HL‐60 Cells

HL‐60 cells were cultured in IMDM+20%FBS, and treated with 1 µm ATRA for 5 days. To assess the rate of HL‐60 differentiation, CD11b expression was detected. Cells were maintained at 37°C and 5% CO_2_ in a humidified incubator (Thermo Fisher, Waltham, MA, USA).

### 4.5 SiRNA Transfection

1 × 10^6^ dHL‐60 cells were placed into a 6‐well plate, and transfected with Si‐NC, Si‐Lcn2, Si‐Cs, Si‐Serpina3, Si‐PTMA, Si‐APMAP, Si‐PSMD11, Si‐C3 as follows: 500 µL Opti‐MEM (GBICO) medium containing 50 nm siRNA was premixed with 5 µL of Lipofectamine RNAiMAX (Thermo Fisher) for 15 min at R.T., then brought up to 2.5 mL by adding additional 2 mL cell suspension, plated and cultured for 48 h before experiments. The successful silencing was confirmed by qPCR. SiRNA Sequences were listed in Table S7.

### 4.6 Induction of Myocardial MI/R Model and Treatments

The mouse myocardial MI/R model was performed as previously reported with slight modifications [54]. In brief, mice were anesthetized with Avertin (Sigma–Aldrich). A small skin cut was made over the left chest and a purse suture was made. After dissection and retraction of the pectoral major and minor muscle, the fourth intercostal space was exposed. A small hole was made at the fourth intercostal space to open the pleural membrane and pericardium. Then the heart was smoothly and gently “popped out” through the hole. The left anterior descending coronary artery (LCA) was located and a slipknot was tied around the LCA with a 6‐0 silk suture. The heart was then quickly placed back into the thoracic space followed by manual evacuation of air and skin closing. After 60 min of ischemia, the slipknot was released by pulling the long end of the slipknot suture smoothly and gently until a feeling of release was sensed. Then mice were randomly divided into each group, including the model group and treatment groups in a blinded manner.

To evaluate the effects of pharmacological NETs inhibition on MI/R injury, mice were pretreated with 75 mg/kg Cl‐amidine hydrochloride or 50 µg DNase I intraperitoneally 24 h before the induction of MI/R. 12 h after reperfusion, mice were again injected with 75 mg/kg Cl‐amidine hydrochloride or 50 µg DNase I again and sacrificed 24 h after reperfusion.

To determine the in vivo function of PLA2G6, we pre‐treated mice with 25 mg/kg Rilapladib intraperitoneally 24 h before the induction of MI/R. 12 h after reperfusion, mice were again injected with 25 mg/kg Rilapladib again and sacrificed 24 h after reperfusion.

### 4.7 Evaluation of Myocardial Infarct Size

The infarct area of the myocardium was determined by Evans blue and 2,3,5‐triphenyltetrazolium chloride (TTC) staining of heart sections. In brief, at the end of the MI/R model, mice were sacrificed, and the LAD was re‐ligated in the same segment with 6‐0 silk suture. 2% Evans Blue (Sigma–Aldrich) was injected by femoral vein while the non‐ischemic area showed blue staining. The heart was then frozen at −20°C for 1 h and was sliced into 3‐mm‐thick transverse sections for about three slices. Then, cardiac slices were incubated with 1% TTC solution (Sigma‐Aldrich) at 37°C for 30 min. The infarcted myocardium appeared white. The percentage of infarct size was calculated as white area (infarcted)/non‐blue area (area at risk) (%) using Image J software.

### 4.8 Histopathological Analysis

Hearts were dissected, washed with PBS for three times, and fixed with 4% paraformaldehyde for 24 h. The hearts were then embedded, sliced into 5‐µm‐thick sections. Deparaffinization of the sections were performed and the sections were then stained with hematoxylin and eosin and Masson's trichrome staining according to standard procedures.

TUNEL staining was performed to identify cardiomyocytes apoptosis. Briefly, heart sections were deparaffinization and detected using One‐step TUNEL In Situ Apoptosis Kit (Green, FITC) (Elabscience, Wuhan, China) following the manufacturer's instructions. TUNEL positive cells (%) were calculated as (number of TUNEL positive cells) / (total cells) ×100% using Image J software.

### 4.9 Caspase Activity Detection

20 mg fresh cardiac tissues were homogenized with lysis buffer and the supernatants were collected. Protein concentration was determined by the BCA assay. The relative activity of each caspase including caspase‐1, caspase‐3, caspase‐9, caspase‐8, and caspase‐11 was determined by Caspase 1/3/8/9/11 Activity Assay Kit (Beyotime Biotechnology, Nantong, China) following the manufacturer's instructions and normalized by protein concentration of each sample.

### 4.10 Cytokines Detection

Concentration of IL‐1β, IL‐6, and HMGB1 in cardiac tissues were detected by ELISA (Excell Bio, Suzhou, China) following the manufacturer's instructions.

### 4.11 Immunofluorescence Detection

To detect the infiltration of neutrophils and NETs formation in vivo, 5‐µm‐thick heart sections underwent deparaffinization and antigen retrieval using citrate buffer solution. Tissue sections were then blocked by 3% BSA solution for 1 h at room temperature, followed by incubation of primary antibodies overnight at 4°C as follows: Anti‐Histone H3 (citrulline R2+R8+R17) antibody (ab5103, 1:250), Anti‐MPO antibody (AF3667, 1:200), Neutrophil Elastase (ELANE) Rabbit mAb (A8953, 1:250), Cleaved Gasdermin D (#10137, 1:250). Donkey anti‐Rabbit IgG (H+L) Highly Cross‐Adsorbed Secondary Antibody, Alexa Fluor 555 (Thermo Fisher Scientific, 1:500), and FITC Rabbit Anti‐Goat IgG (H+L) (Abclonal, 1:500) were used as secondary antibody. Nucleus were stained using Hoechst 33342 (Beyotime, P0133, 1:1000). Cell images were acquired on confocal microscopy LSM700 (Zeiss, Oberkochen, Germany).

### 4.12 Immune Cells Detection by Flow Cytometry

Cardiac immune cell populations were detected by flow cytometry as previously reported [55]. Briefly, mice were euthanized and hearts were perfused with 20 mL of cold PBS using a 20 mL syringe fitted with a 21G needle. Then the hearts were dissected, minced using curved dissecting scissors, digested with 1 mL heart digestion buffer (DMEM containing 450 U/ml collagenase I (Invitrogen), 60 U/ml hyaluronidase type I‐S (Sigma–Aldrich), and 60 U/ml DNase I (Sigma‐Aldrich)) at 37°C for 60 min. Samples were filtered through 70 µm cell strainer and centrifuged at 400 g for 5 min at 4°C. Red blood cells were lysed by adding 1 mL of RBC lysis buffer (Biolegend). Samples were resuspended with PBS and blocked with anti‐mouse CD16/32 antibody (Biolegend) at room temperature for 10 min. Then the cells were stained with Zombie NIR Fixable Viability Kit for 10 min at room temperature. Samples were washed with PBS and resuspended with 200 µL FACS buffer. Cells were stained with APC anti‐mouse Ly6C antibody, Percp‐Cy5.5 anti‐mouse Ly6G antibody, Brilliant Violet 605 anti‐mouse/human CD11b antibody, Pacific Blue anti‐mouse CD45 antibody, Alexa Fluor 700 anti‐mouse CD11c antibody, Brilliant Violet 510 anti‐mouse CD103 antibody, and PE anti‐mouse F4/80 antibody. Cells were washed with PBS and resuspended with 500 µL PBS. Data were collected by BD FACS Celesta (BD) and analyzed by Flowjo V10 software. T‐SNE plots were generated by Flowjo V10 software.

### 4.13 Label‐Free Proteomics Analysis

Protein extraction, digestion, and desalination: Fresh cardiac tissues (about 30 mg) were collected, frozen, and stored at ‐80°C. 300 µL of 8 m urea was added to the sample. After centrifuging at 14 100×g for 20 min, the supernatant was collected. The protein concentration was determined using Bradford method. 100 µg protein from each sample was then subjected to a reduction reaction by adding 200 mm dithiothreitol (DTT) solution and incubating at 37°C for 1 h. The sample was diluted 4 times by adding 25 mm ammonium bicarbonate (ABC) buffer, then added trypsin (trypsin: protein = 1:50) and incubating at 37°C overnight. 50 µL 0.1% FA was added to terminate the digestion. Peptides were desalted using C18 column.

LC‐MS/MS Analysis: Nanoflow LC‐MS/MS analysis of tryptic peptides was conducted on a quadrupole Orbitrap mass spectrometer (Orbitrap Fusion, Thermo Fisher Scientific, Bremen, Germany) coupled to an EASY nLC 1200 ultra‐high pressure system (Thermo Fisher Scientific) via a nano‐electrospray ion source. 500 ng of peptides were loaded on a 25 cm column (150 µm inner diameter, packed using ReproSil‐Pur C18‐AQ 1.9‐ µm silica beads; Beijing Qinglian Biotech Co.,Ltd, Beijing, China). Peptides were separated using a gradient from 3% to 8% B in 8 to 11 min, then 8% to 20% B in 77 min and stepped up to 40% in 10 min followed by a 15 min wash at 90% B at 600 nL per minute where solvent A was 0.1% formic acid in water and solvent B was 80% ACN and 0.1% formic acid in water. The total duration of the run was 60 min. Column temperature was kept at 60°C using an in‐house‐developed oven. Briefly, the mass spectrometer was operated in “top‐40” data‐dependent mode, collecting MS spectra in the Orbitrap mass analyzer (120 000 resolution, 250–1450 m/z range) with a maximum ion injection time of 50 ms. The most intense ions from the full scan were isolated with an isolation width of 1.6 m/z. Following higher‐energy collisional dissociation (HCD) with a normalized collision energy (NCE) of 30, MS/MS spectra were collected in the Orbitrap (30 000 resolution) with a maximum ion injection time of 50 ms. Precursor dynamic exclusion was enabled with a duration of 16 s.

Data analysis: All RAW files were analyzed using the Proteome Discoverer suite (version 2.4, Thermo Fisher Scientific). MS2 spectra were searched against the UniProt Mouse proteome database containing both Swiss‐Prot and TrEMBL Mouse reference protein sequences (55 338 target sequences downloaded on 13 August 2020). The Sequest HT search engine was used, and parameters were specified as follows: fully tryptic specificity, maximum of two missed cleavages, minimum peptide length of 6, fixed carbamidomethylation of cysteine residues (+57.02146 Da), variable modifications for oxidation of methionine residues (+15.99492 Da), precursor mass tolerance of 15 ppm and a fragment mass tolerance of 0.02 Da for MS2 spectra collected in the Orbitrap. Percolator was used to filter peptide spectral matches and peptides to a false discovery rate (FDR) of less than 1%. After spectral assignment, peptides were assembled into proteins and were further filtered based on the combined probabilities of their constituent peptides to a final FDR of 1%. As default, the top matching protein or “master protein” was the protein with the largest number of unique peptides and with the smallest value in the percent peptide coverage (that was, the longest protein). Only unique and razor (that was, parsimonious) peptides were considered for quantification. Proteomic determination and data analysis were carried out with the assistance of Beijing Qinglian Biotech Co.,Ltd, Beijing, China.

### 4.14 Neutrophil Depletion in Mice

Mice were intraperitoneally injected with 100 µg of InVivoMAb anti‐mouse Ly6G (Bio X cell, clone 1A8) or with InVivoMAb rat IgG2α isotype control, anti‐trinitrophenol (Bio X cell, clone 2A3). The percentage of neutrophils in peripheral blood and cardiac tissue was analyzed by flow cytometry after 24 h. Mice were then subjected into MI/R model as mentioned above.

### 4.15 Adoptive Transfer of Neutrophils

Adoptive transfer of *Gsdmd* ^−/−^ neutrophils were performed as previously reported with slight modification [56]. Briefly, CD45.1 recipient mice were injected with anti‐Ly6G neutralizing antibody 24 h before induction of MI/R injury as described above. Neutrophils were purified using Neutrophil Isolation Kit, mouse (130‐097‐658, Miltenyi Biotec, San Diego, CA, USA). 8 × 10^6^ neutrophils from CD45.2 *Gsdmd* ^−/−^ mice and were transferred into CD45.1 recipient mice by intravenous injection 12 h before MI/R injury. The chimerism were confirmed by flow cytometry.

### 4.16 qPCR Analysis

Total RNA was extracted from ischemic heart or neonatal cardiomyocytes using RNA isolator Reagent (Vazyme, Nanjing, China) according to the manufacturer's instructions. RNA was converted to cDNA using HiScript III RT SuperMix for qPCR (Vazyme). Real‐time PCR was performed on CFX96 (Bio‐Rad, Hercules, CA, USA). *Actb* was used as an internal control gene. Primers used were listed in Table S8.

### 4.17 Quantification of Serum Cell‐Free dsDNA and MPO‐DNA Nucleosomes

Serum cell‐free dsDNA (cfDNA) was quantified by Equalbit dsDNA HS Assay Kit (#EQ111‐01, Vazyme) following the manufacturer's instructions. MPO‐DNA complexes were determined as previously reported [57]. Briefly, plates were coated with 2 µg/mL anti‐mouse MPO antibody (NOVUS Biologicals) overnight at 4°C. Then the plates were washed with wash buffer for three times followed by blocked with 1% BSA in PBS for 2 h at room temperature. The plates were then washed three times, 50 µL serum samples were added to each well and incubated for 1 h at room temperature. The plates were washed five times and incubated with anti‐DNA antibody (HRP‐conjugated) from the Cell Death Detection ELISA kit (Roche, Saint Louis, MO, USA) for 2 h. Plates were then washed five times and TMB substrate was added. The stop solution was then added. Absorbance was measured at 450 nm using a Synergy H1 multi‐mode reader (BioTek, Winooski, VT, USA).

### 4.18 Isolation of Primary Neutrophils and Cardiomyocytes

Primary neutrophils were isolated using percoll density centrifugation [58]. Briefly, bone marrow cells were flushed out by HBSS (without Ca^2+^ and Mg^2+^) from two femurs and two tibias using a 5 mL syringe and a 25G needle. After centrifuge at 230 g for 6 min at room temperature, the supernatant was discarded and 3 mL HBSS was added. 52%, 64% and 72% Percoll density solution were prepared. Layer the bone marrow suspension gently on the top of 54% Percoll solution. Centrifuge the gradient at 1550 g for 30 min at room temperature without a brake. Then carefully remove the first and second cell layers and transfer the cell layer between 64% and 72% into a clean 15 mL conical tube. Wash cells with 5 mL HBSS and resuspended in 1640. Cells were counted and seeded into 96‐well plates or 6‐well plates.

Primary cardiomyocytes were isolated from neonatal mice as described [59]. Briefly, neonatal mice (day 0) were decapitated using sterile scissors without anesthesia, and chests along the sternum were opened. Nearly 6–8 hearts were mixed, residual lung tissue and larger vessels were removed and each heart was cut into 8 pieces. Hearts were then pre‐digested with 0.5 mg/mL Trypsin and further digested with Collagenase solution (350 U/L collagenase Type II) at 37°C for 10 min twice. Cells were then passed through a 100 µm cell‐strainer and centrifuged at 180 g for 5 min. Gently resuspended cells with 10 mL DMEM and plate cells onto a 10 cm cell culture dish and incubated for 1 h. After 1 h incubation, gently agitated the plate and wash non‐adherent cells (enriched in cardiomyocytes) and transferred into a new 10 cm cell culture dish and incubated for an additional 1 h. Wash non‐adherent cells from the 10 cm culture dish and count the cells. Cells were seeded into plates coated with PBS containing 0.5% gelatin and 1% fibronectin and analyzed after 2 days.

### 4.19 Endogenous Metabolite Library and FDA‐Approved Drug Library Screening

To screen endogenous metabolites by Sytox green high throughput screening, primary neutrophils were isolated and seeded in 96‐well plate (1 × 10^5^/well) as mentioned above. Each metabolite was added to a final concentration of 40 µm and incubated for 3 h. 100 nm Sytox green DNA probe (Thermo Fisher Scientific) were added and incubated at room temperature for 15 min. Fluorescence intensity was measured at Ex 488 nm, Em 525 nm using Synergy H1 multi‐mode reader (BioTek, Winooski, USA). Relative fluorescence intensity was normalized by vehicle control group. PMA serves as a positive control on each plate.

To screen endogenous metabolites by high content imaging, primary neutrophils were isolated and seeded in 96‐well plate (1 × 10^5^/well) as mentioned above. Each metabolite was added to a final concentration of 40 µm and incubated for 3 h. Cells were fixed by paraformaldehyde, permeabilized by 0.1% Triton X and blocked by 3% BSA. Then cells were stained with Goat anti‐MPO antibody (NOVUS, 1:200), rabbit anti‐Histone H3 (citrulline R2 + R8 + R17) (Abcam, 1:250) overnight. Cells were washed with PBST and stained with Donkey anti‐Rabbit IgG (H+L) Highly Cross‐Adsorbed Secondary Antibody, Alexa Fluor 555 (Thermo Fisher Scientific, 1:500), and FITC Rabbit Anti‐Goat IgG (H+L) (Abclonal, 1:500) for 2 h at room temperature. Nucleus were stained with Hoechst (Beyotime). High content imaging was conducted by Opera Phenix (Perkin Elmer, Germany). The percentage of H3Cit positive cells were calculated.

To screen NETosis inhibitor, primary neutrophils were isolated and seeded in 96‐well plate (1 × 10^5^/well) as mentioned above. A total of 1499 FDA‐approved drugs were then added to a final concentration of 20 µm and incubated for 1 h. 100 nm PMA or 5 µm PAF was added to trigger NETosis for 3 h. 100 nm Sytox green DNA probe (Thermo Fisher Scientific) were added and incubated at room temperature for 15 min. Fluorescence intensity was measured at Excitation 488 nm, Emission 525 nm using Synergy H1 multi‐mode reader (BioTek). PMA or PAF were set on each plate and considered as 100% cell death. The relative cell death rate of each compound was calculated.

### 4.20 Lip‐MS Analysis

Primary mouse bone marrow‐derived neutrophils were isolated and treated with DMSO, 800 µm dapagliflozin, and 800 µm ertugliflozin for 6 h, Liquid nitrogen was added to sample and added appropriate amount of Native buffer (lysate: protease inhibitor 50:1). After centrifuging at 14 000×g for 20 min, the supernatant was collected. The protein concentration was determined using the Bradford method, rest was frozen at −80°C.

The protein of each sample was divided into 2 parts, and two kinds of enzyme digestion were performed: 1) protease K+ Tyrisin (LiP group); 2) Only Tyrisin (TrP group). In the LiP group, protease K was added into the protein solution according to m(sample protein):m(protease K) = 100:1, incubated at 25°C for 1 min, treated at 100°C for 5 min immediately to terminate the enzyme digestion, and cooled to room temperature. In all samples (TrP group and LiP group), SDC was added to denature the proteins, and reduced alkylation was performed with TCEP. IAA was added and incubated at room temperature for 1 h away from light. Adjust pH to 8–9, add Lys according to m(sample protein): m(Lys) = 100:1, and incubate at 37°C for 4 h; Dilute the SDC in the sample to 1%, adjust the pH to 7–9, add Trypsin according to m(sample protein):m(Trypsin) = 100:1, and perform overnight enzyme digestion at 37°C. After digestion, FA was added to adjust the pH of the system < 3. Supernatant was obtained by centrifugation at 4°C at 16 000 g for 10 min. The sample solution was dried after desalting, stored at −80°C or directly subjected to subsequent mass spectrometry.

Nanoflow LC‐MS/MS analysis of tryptic peptides was conducted on a quadrupole Orbitrap mass spectrometer (Q Exactive HF‐X, Thermo Fisher Scientific, USA) coupled to an EASY nLC 1200 ultra‐high pressure system (Thermo Fisher Scientific) via a nano‐electrospray ion source.1ug of peptides were loaded on a 25 cm column (100 µm inner diameter, packed using ReproSil‐Pur C18‐AQ 1.5‐ µm silica beads; QL‐HPLC‐100×15; Beijing Qinglian Biotech Co., Ltd, Beijing, China). Peptides were separated using a gradient from 8% to 12% B in 8 min, then 12% to 30% B in 55 min and stepped up to 40% in 12 min followed by a 15 min wash at 95% B at 300 nL per minute where solvent A was 0.1% formic acid in water and solvent B was 80% ACN and 0.1% formic acid in water. The total duration of the run was 90 min. Column temperature was kept at 60°C using an in‐house‐developed oven. Briefly, the mass spectrometer was operated in “top‐40” data‐dependent mode, collecting MS spectra in the Orbitrap mass analyzer (120 000 resolution, 350–1500 m/z range) with an automatic gain control (AGC) target of 3E6 and a maximum ion injection time of 80 ms. The most intense ions from the full scan were isolated with an isolation width of 1.6 m/z. Following higher‐energy collisional dissociation (HCD) with a normalized collision energy (NCE) of 27, MS/MS spectra were collected in the Orbitrap (15 000 resolution) with an AGC target of 5E4 and a maximum ion injection time of 45 ms. Precursor dynamic exclusion was enabled with a duration of 16 s.

All RAW files were analyzed using the Proteome Discoverer suite (Thermo Fisher Scientific). MS2 spectra were searched against the UniProtKB Mouse proteome database containing both Swiss‐Prot mouse reference protein sequences. The Sequest HT search engine was used, and parameters were specified as follows: Semi‐Tryptic, maximum of two missed cleavages, minimum peptide length of 6, fixed carbamidomethylation of cysteine residues (+57.02146 Da), variable modifications for oxidation of methionine residues (+15.99492 Da), precursor mass tolerance of 15 ppm and a fragment mass tolerance of 0.02 Da for MS2 spectra collected in the Orbitrap. Percolator was used to filter peptide spectral matches and peptides to a false discovery rate (FDR) of less than 1%. After spectral assignment, peptides were assembled into proteins and were further filtered based on the combined probabilities of their constituent peptides to a final FDR of 1%. As default, the top matching protein or “master protein” was the protein with the largest number of unique peptides and with the smallest value in the percent peptide coverage (that was, the longest protein). Only unique and razor (that was, parsimonious) peptides were considered for quantification.

### 4.21 Molecular Docking

In the target identification, five crystal structures and two AF2 structures were collected for the analysis, including PDB structures for CS (5uzp), SerpinA3k (9d7k), PSMD7 (5vfp), CO3 (6ru5), and LCN2 (3hwe), and AF2 structures for PTMA (AF‐P06454‐F1‐v4) and APMAP (AF‐Q9HDC9‐F1‐v4). Autodock Vina/1.1.2 was used for molecular docking. The docking box was set with 20 Å × 20 Å × 20 Å centered at the co‐ligand binding site of each target. All the docking simulations were performed for 20 times, with the top‐1 docking pose employed for the analysis.

### 4.22 Statistical Analysis

Data are presented as mean ± SEM, the number of biological replicates for each experiment is indicated in figure legends. Graphs and all statistical analysis were performed using GraphPad Prism (version 8.0), and quantifications of infarct size, fibrotic area, and netting neutrophils (H3Cit^+^% cells) were conducted by Image J. Flow cytometry analysis was carried out using BD Accuri C6 plus and BD FACS Celesta and further analyzed by Flowjo V10 software. For high‐content image screening, data were acquired by high‐content imaging system (Opera Phenix, PerkinElmer, USA) and analyzed by Harmony 4.9 software. The serum biochemical indicators in human subjects were analyzed by Cobas 6000 (Roche). For image data, one representative image of three independent experiments is shown. The researchers were not blinded and no statistical methods were used to predetermine sample size. For experiments with two groups, statistical analysis was performed with Student's *t*‐test with 95% confidence interval. For experiments with more than two groups, a one‐way ANOVA with Turkey post hoc analysis was performed, *p* values under 0.05 were considered statistically significant, otherwise indicated as nonsignificant (n.s).

## Author Contributions

Contribution: L.C. and H.H. conceptually designed the study, wrote and revised the manuscript, H.S. was responsible for molecular docking, and supervised target validation data. J.W., S.Z., and R.D. performed most of the experiments and analyzed the data. L.K. and X.B. were responsible for clinical translational validation experiments. Z.X. and T.H. contributed to partial experiments and help to analyze the data. J.W., S.Z., R.D., and L.K. contributed equally to this work. All authors have read and approved the final manuscript.

## Funding

This work was financially supported by the National Key Research and Development Programme of China (2021YFA1301300 and 2022YFF1100601), National Natural Science Foundation of China (82373886 and 82321005), Overseas Expertise Introduction Project for Discipline Innovation (G20582017001), CAMS Innovation Fund for Medical Sciences (SZSM202301035), Jiangsu Provincial Outstanding Youth Science Foundation Project (BK20250097) and the Natural Science Foundation of Jiangsu Province (BK20253058).

## Conflicts of Interest

The authors declare no conflicts of interest.

## Supporting information




**Supporting file**: advs74533‐sup‐0001‐SuppMat.docx

## Data Availability

All data generated or analyzed in this study are available from the corresponding author upon reasonable request (contact to Lijuan Cao, caolijuan0702@cpu.edu.cn). The mass spectrometry proteomics data of cardiac tissues and Lip‐MS data have been deposited to the ProteomeXchange Consortium (http://proteomecentral.proteomexchange.org) via the iProX partner repository with the dataset identifier PXD040135 and PXD062892, respectively.
