# Supplementary material for: PAF Triggered Pyroptotic NETosis Aggravates Myocardial Ischemia/Reperfusion Injury
Source: Adv Sci (Weinh). 2026 Feb 25;13(25):e19140. doi: 10.1002/advs.202519140 (PMC13137845; doi:10.1002/advs.202519140)
Supplement: Supplementary file 1 — Supporting file: advs74533‐sup‐0001‐SuppMat.docx [file ADVS-13-e19140-s001.docx]

Supplementary information

**PAF triggered pyroptotic NETosis aggravates myocardial ischemia/reperfusion injury**

Jiawei Wu^1, 4,^ ^#^, Shule Zhang^1, 2, #^, Ruofan Du^1, 2 #^, Lina Kang^3, #^, Guodong Zhao^1, 2^, Xue Bao^3^, Haochi Yang^1, 2^, Ziqing Xie^1, 2^, Tianyu He^1, 2^, Huiyong Sun^2, *^, Haiping Hao^1, 2, *^ and Lijuan Cao^1, 2*^

^1^ State Key Laboratory of Natural Medicines, China Pharmaceutical University, Nanjing, P. R. China.

^2^ Jiangsu Provincial Key Laboratory of Targetome and Innovative Drugs, Institute of Innovative Drug Discovery and Development, China Pharmaceutical University, Nanjing, P. R. China.

^3^ Department of Cardiology, Nanjing Drum Tower Hospital Affiliated to Nanjing University Medical School, Nanjing, China.

^4^ Department of Gastroenterology, Kunshan Hospital Affiliated to Jiangsu University, Kunshan 215300, China.

^#^ These authors contributed equally to this work.

^*^ Corresponding author e-mail: [caolijuan0702@cpu.edu.cn](mailto:caolijuan0702@cpu.edu.cn) (Lijuan Cao); [haipinghao@cpu.edu.cn](mailto:haipinghao@cpu.edu.cn) (Haiping Hao); huiyongsun@cpu.edu.cn (Huiyong Sun).

# This file includes:

Figs. S1 to S11

Tables S1 to S8


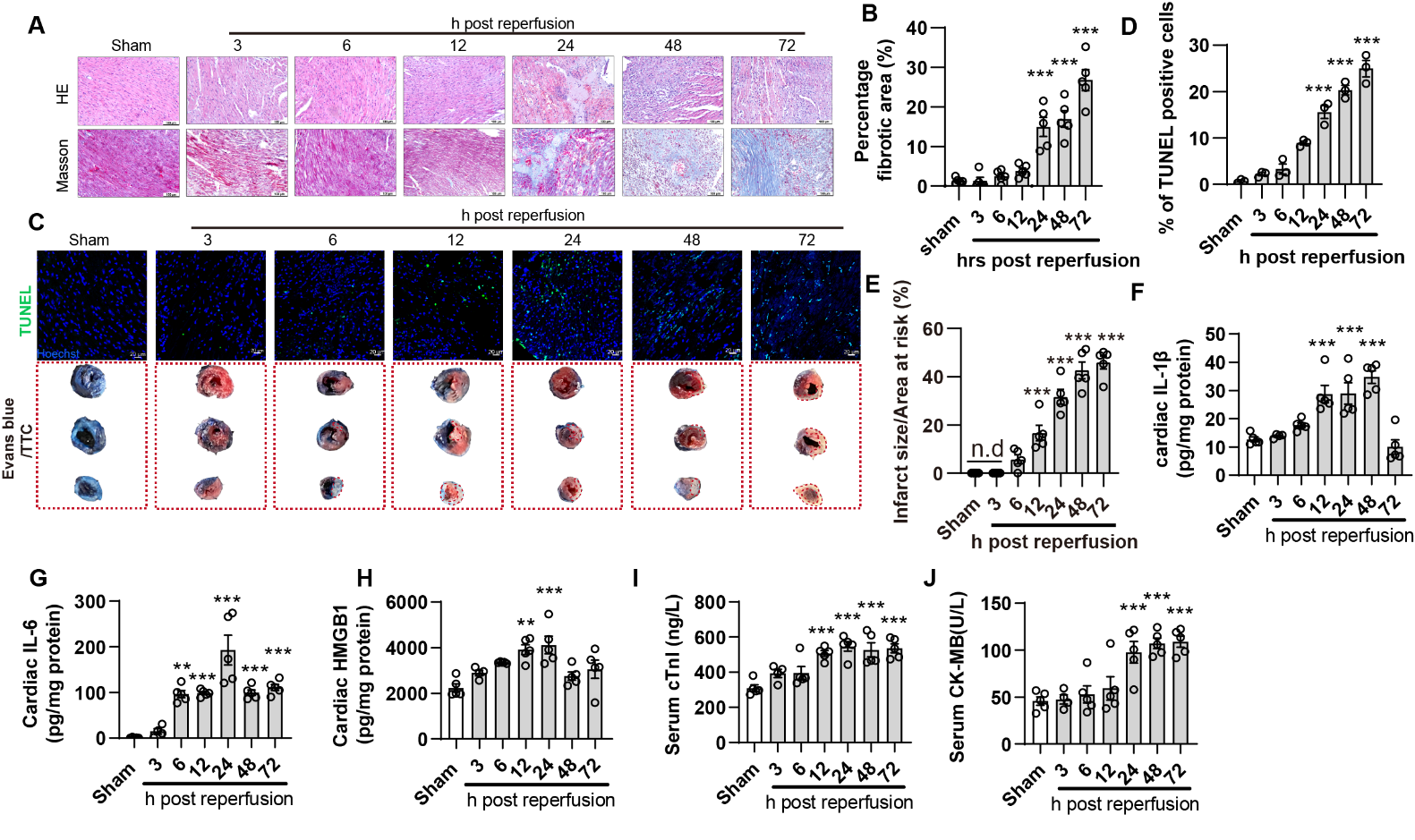


**Fig. S1. Changes of cardiac injury during MI/R injury.**Left anterior descending coronary artery (LCA) of mice were ligated for 60min to induce ischemia, then the slipknot is released for indicated time to induce reperfusion (n=5, otherwise indicated). **(A)** Representative image of HE staining (upper panel) and Masson’s trichrome staining (lower panel) of heart section at different time points. Scale bar, 100 μm. **(B)** Percentage of fibrotic area in each group, quantified data from (A). **(C)** Representative TUNEL staining (upper panel) and TTC/Evans Blue staining (lower panel) indicate cell death during MI/R injury. Green, TUNEL probe. Blue, Hoechst 33342. Scale bars, 20 μm. **(D)** TUNEL positive cells, quantified from TUNEL staining (**C**) (n=3). **(E)** Percentage of infarct size within area at risk, quantified from TTC/Evans Blue staining (**C**). (**F-H**) ELISA analysis of IL-1β (**F**), IL-6 (**G**) and HMGB1 (**H**) in cardiac tissues (n=4-6). (**I**) Serum cTnI level. (**J**) Serum CK-MB level. Data are presented as mean±SEM and normalized by cardiac protein concentration. ***P* < 0.01, ****P* < 0.001 compared with sham group.

**
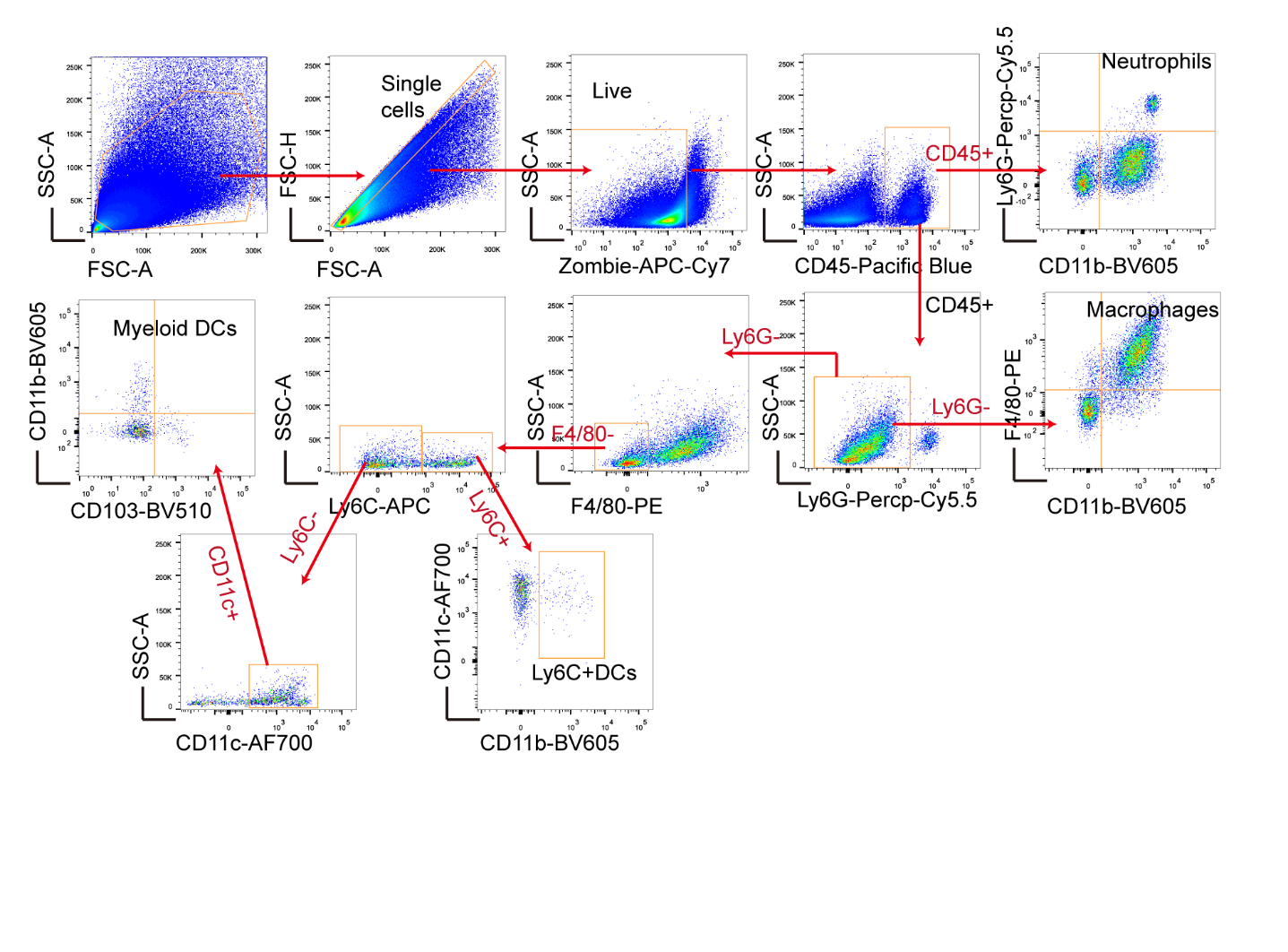
**

**Fig. S2. Gating strategy of immune cell populations in heart.**

**
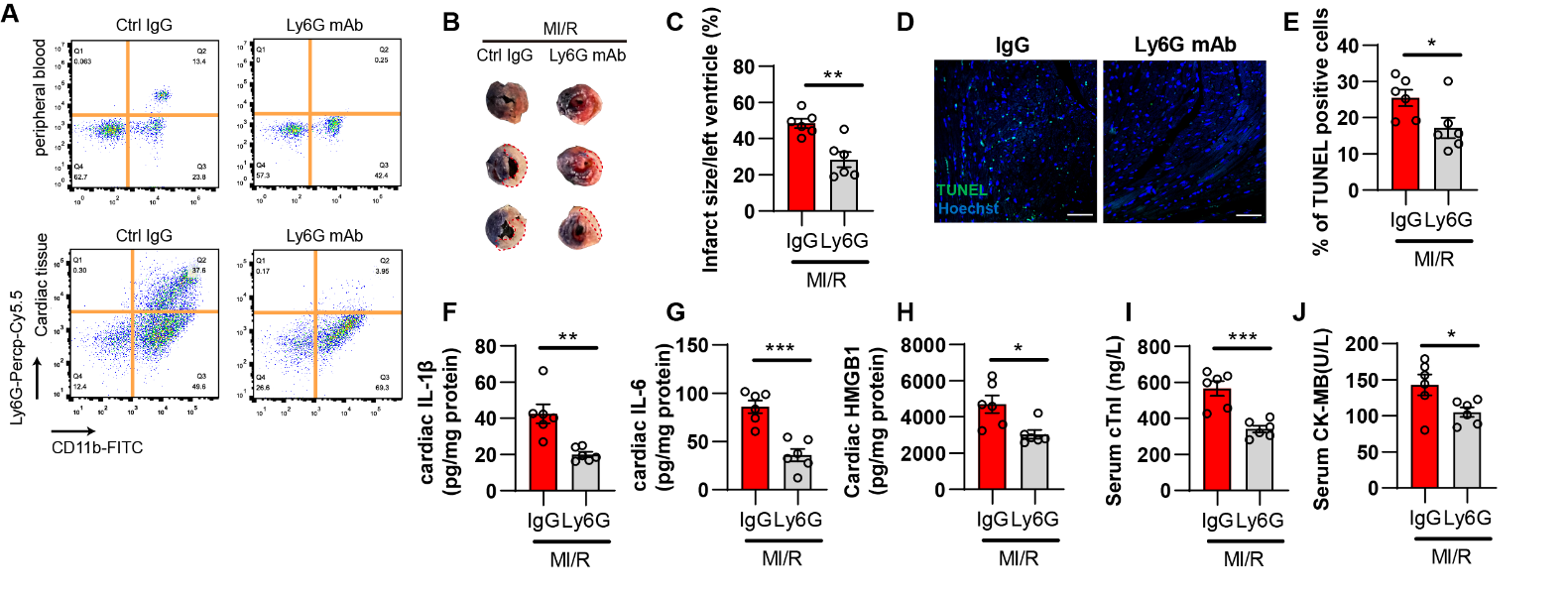
**

**Fig. S3. Depletion of neutrophils mitigates MI/R injury. (A)** Flow cytometry analysis of neutrophils in blood and cardiac tissues after administration of Ly6G neutralizing antibody or Ctrl IgG. Representative images were shown. **(B-E)** Mice receiving Ctrl IgG or Ly6G mAb were subjected into MI/R injury, TTC-Evan Blue staining (**B**), infarct size (**C**), TUNEL analysis (**D**) and quantification of TUNEL positive cells (**E**) were shown (n=6). Red dotted line indicates infarct area. (**F-H)** Cardiac level of IL-1β (**F**), IL-6 (**G**) and HMGB1 (**H**) determined by ELISA (n=6). (**I**) Serum cTnI level. (**J**) Serum CK-MB level. **P* < 0.05, ***P* < 0.01, ****P* < 0.001 compared to Ctrl IgG group. Data are shown as mean ± SEM. Data were analyzed by two-tailed Student’ *t* test of indicated groups.


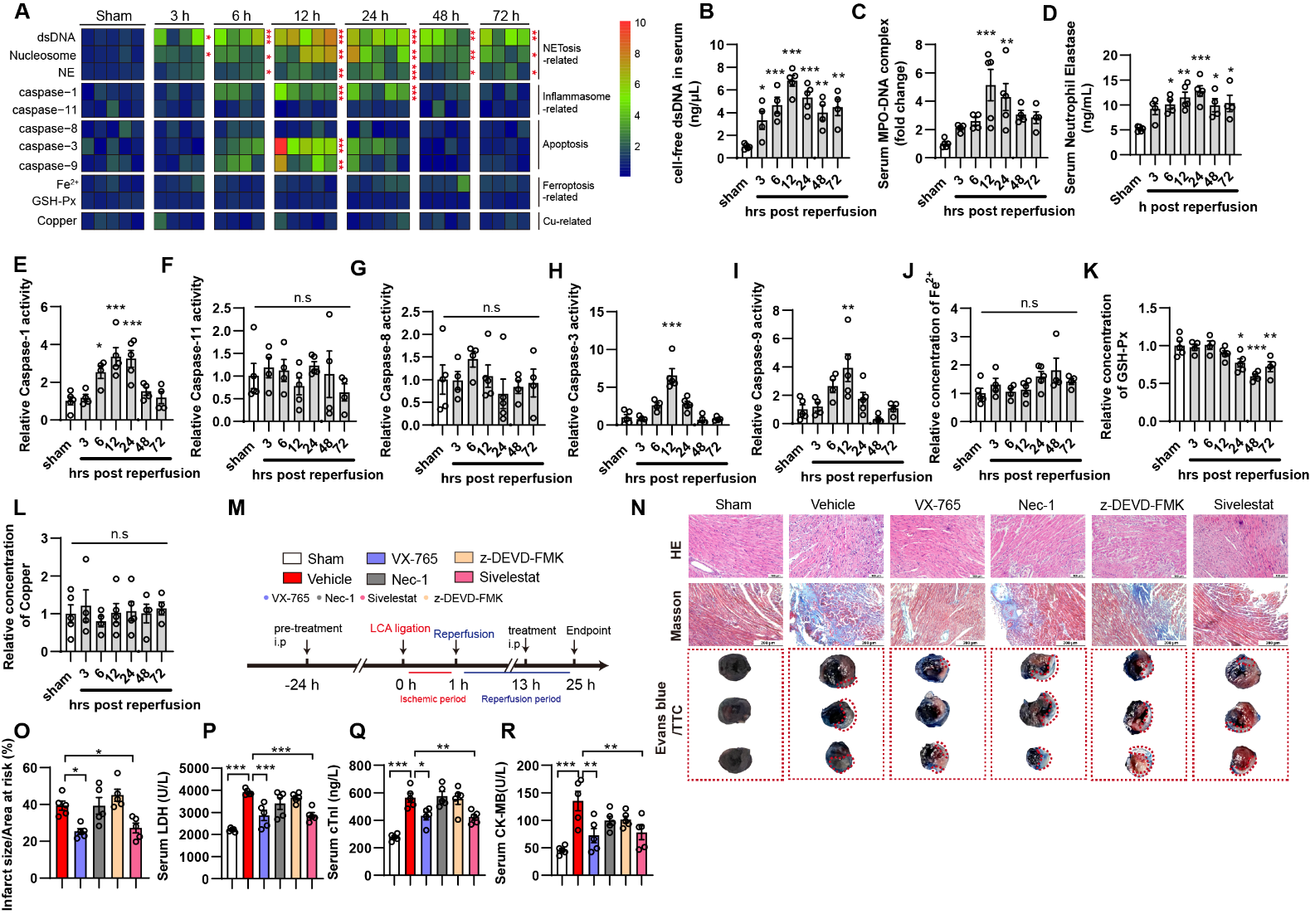


**Fig. S4. NE-mediated NETosis is responsible for MI/R injury.** Animal treatment as described in Figure 1 (n=5, otherwise indicated). (**A-L**) Determination of relative activity of caspase-8/caspase-3/caspase-9, caspase-11/caspase-1, relative concentration of Fe^2+^ and GSH-Px, relative concentration of copper and relative expression of Neutrophil elastase/cell-free dsDNA/MPO-DNA nucleosome complex, which represent apoptosis, inflammasome-related cell death, ferroptosis, curroptosis and NETosis, respectively (n=4-5). **P* < 0.05, ***P* < 0.01, ****P* < 0.001 compared to sham group. (**M**) Schematic illustration of experimental design. Mice were intraperitoneally pre-treated with 50 mg/kg VX-765, 2 mg/kg Nec-1, 20 mg/kg Sivelestat and 5 mg/kg z-DEVE-FMK 24 h before MI/R induction, respectively. A secondary administration of each inhibitor was conducted 12 h after reperfusion. (**N**) Representative images of HE staining (upper panel, scale bar, 100 μm), Masson’s trichrome staining (middle panel, scale bar, 200 μm) and TTC/Evans Blue staining (lower panel) were shown. (**O**) Percentage of infarct size within area at risk (n=5). (**P**) Serum LDH level (n=5). (**Q**) Serum cTnI level (n=5). (**R**) Serum CK-MB level (n=5). **P* < 0.05, ****P* < 0.001 compared to Vehicle group.


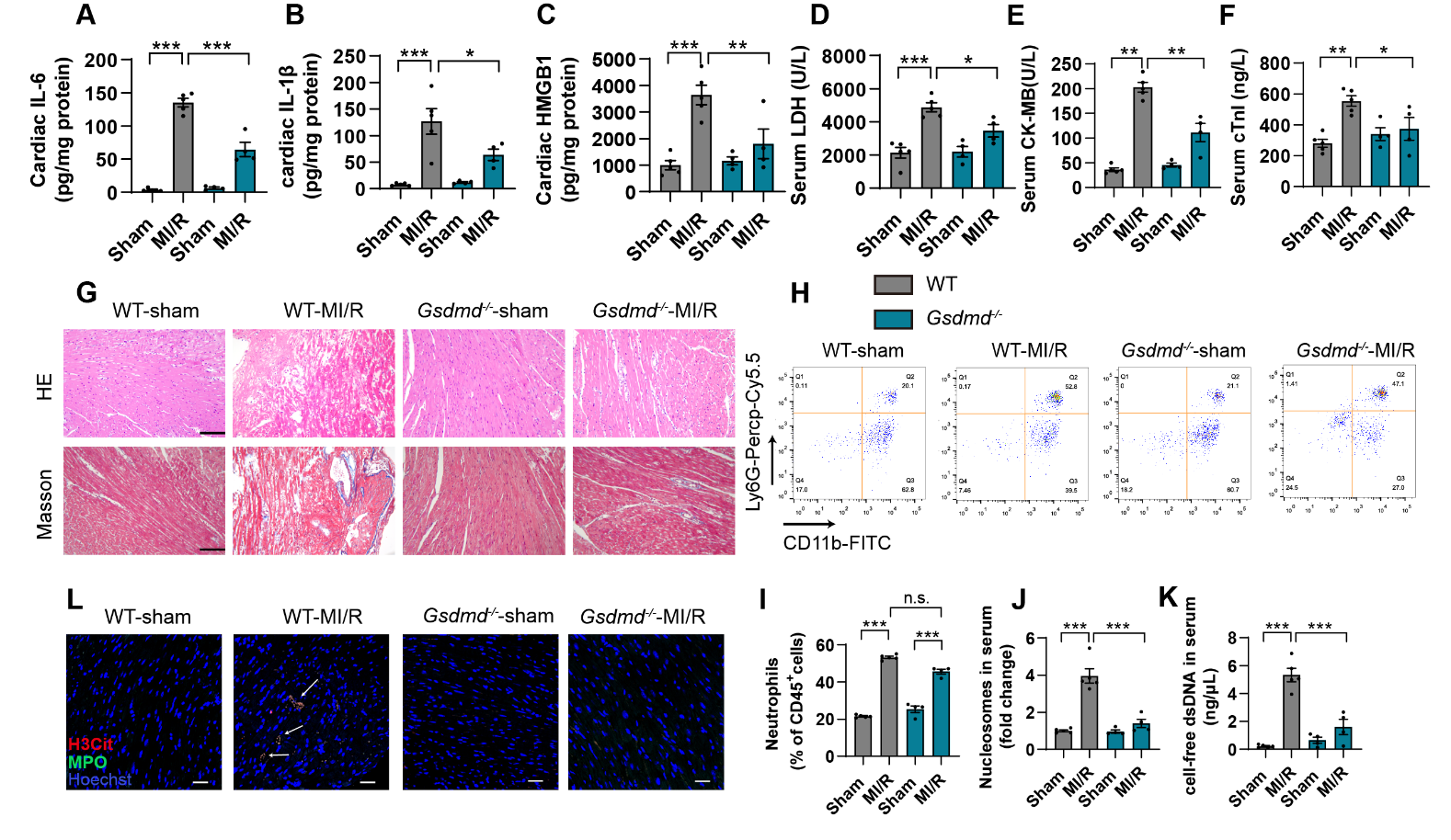


**Fig. S5. *Gsdmd*-knockout protects mice from MI/R injury.** (**A-C**) Cardiac concentration of IL-6 (**A**), IL-1β (**B**) and HMGB1 (**C**) determined by ELISA. (**D**) Serum LDH level. (**E**) Serum CK-MB level. (**F**) Serum cTnI level. (**G**) Representative images of HE staining (upper panel) and Masson’s trichrome staining (lower panel) of heart section. Scale bar, 100 μm. (**H**) Flow cytometry analysis of neutrophils in cardiac tissues in WT or *Gsdmd*^-/-^ mice, representative images were shown. (**I**) Percentage of cardiac neutrophils in each group (n=4-5). (**J, K**) Serum MPO-DNA nucleosome complex (**J**) and cell-free dsDNA (**K**) were determined (n=4-5). (**L**) Representative images of heart section with staining for MPO (green), citrullinated Histone H3 (H3Cit, red) and Hoechst (blue). Scale bar, 50 μm. White arrows indicate NETs. Data are shown as mean±SEM, n=4-5 mice per group. **P* < 0.05, ***P* < 0.01, ****P* < 0.001 compared to WT MI/R group. n.s., not significant.

**
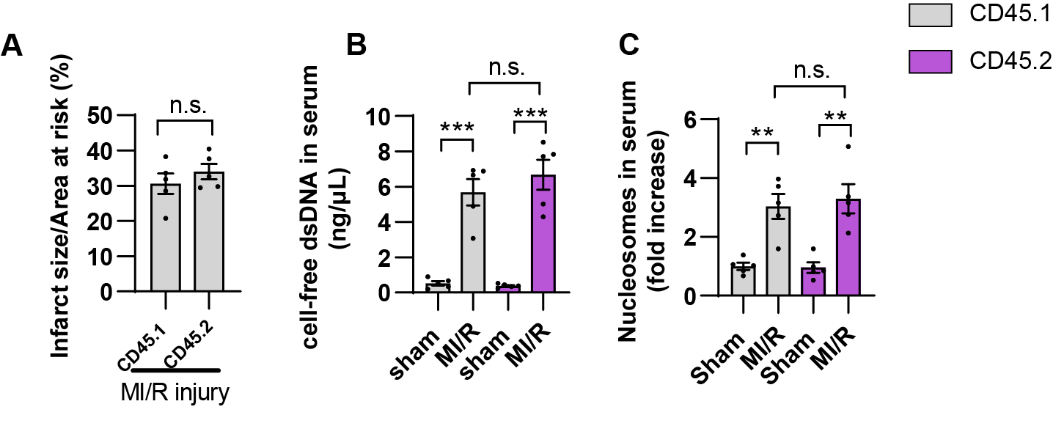
**

**Fig. S6. CD45.1 mice exhibit similar NETosis occurrence and tissue damage during MI/R injury.** (**A**) Quantification of infarct size. (**B and C**) MPO-DNA Nucleosome quantification (**B**) and cell-free dsDNA determination (**C**) in serum. n=5 mice per group. Data are shown as mean±SEM. ***P* < 0.01, ****P* < 0.001 compared to each sham group. n.s., not significant compared to CD45.1 MI/R group.

**
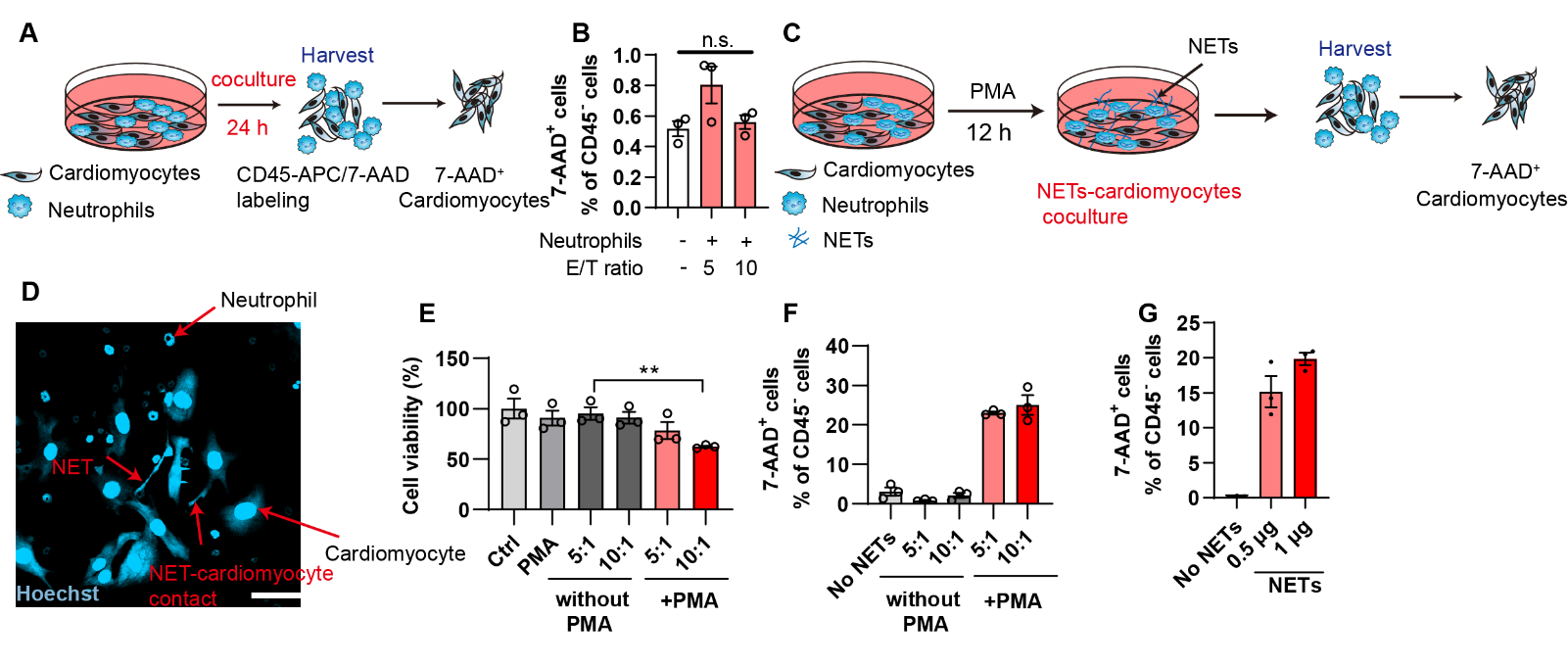
**

**Fig. S7. NETs cause cardiomyocyte death.** (**A**) Experimental design of in vitro cardiomyocyte-neutrophil coculture. Cardiomyocytes were cocultured with primary neutrophils for 24 h. (**B**) Cell death were determined by flow cytometry analysis of the percentage of 7-AAD^+^ cells in CD45^-^ cells. (**C**) Cardiomyocytes were cocultured with neutrophils at indicate ratios, 100 nM PMA were added to trigger NETosis for 12 h. (**D**) Representative immunofluorescence image during the coculture. Nucleus (Cyan) were labelled with Hoechst. Scale bar, 50 μm. (**E**) Cell viability determined by CCK-8 assay (n=3). (**F**) Percentage of 7-AAD^+^ cells in CD45^-^ cells were shown (n=3). (**G**) Primary neutrophils were isolated and stimulated with 100 nM PMA for 3 h. NETs were collected and quantified. Primary cardiomyocytes were treated with indicated concentration of NETs for 12 h. Cell death were determined by flow cytometry (n=3). Data are shown as mean±SEM. ***P* < 0.01. n.s., not significant.

**
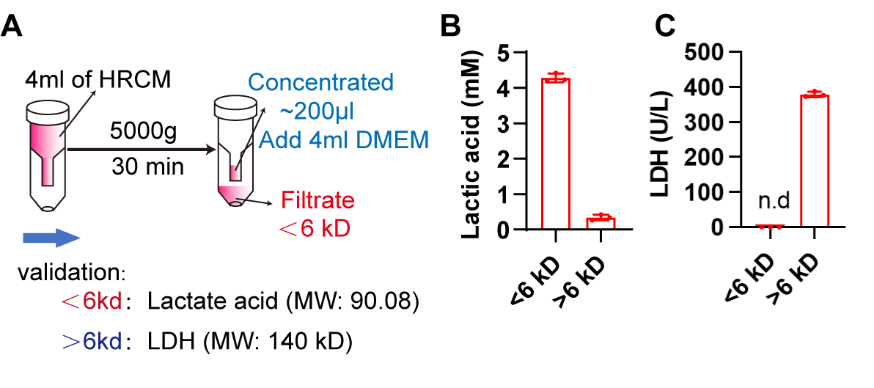
**

**Fig. S8. Identification of ultrafiltration.** (**A**) Illustration of ultrafiltration workflow. (**B and C**) Lactic acid (**B)** and LDH (**C**) measurements (n=3). Data are shown as mean±SEM. n.d, not detected.


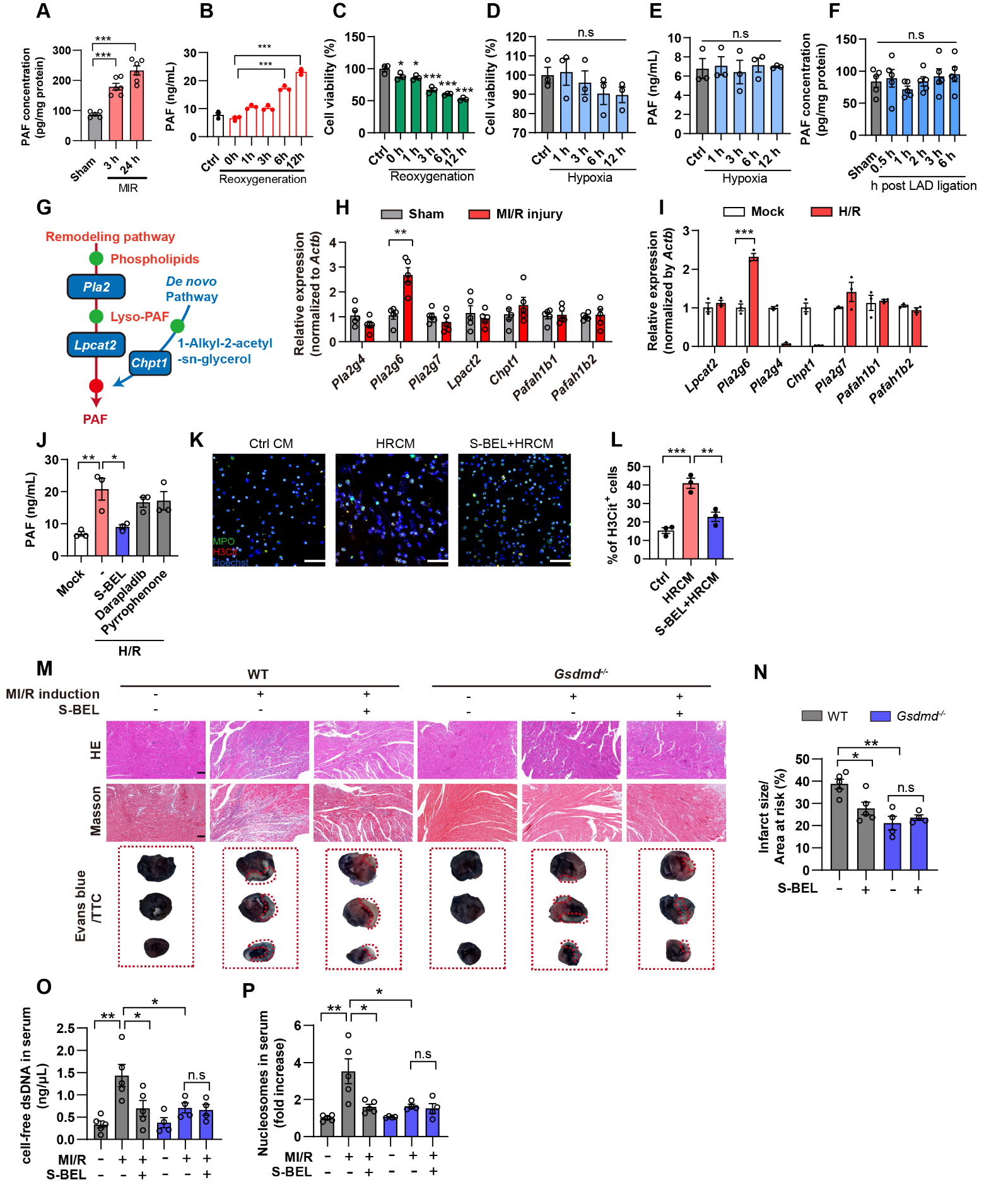


**Fig. S9. Upregulation of PLA2G6 is responsible for excessive PAF production.** (**A**) Cardiac PAF concentration determined by ELISA (n=5-6). ****P* < 0.001 compared to sham group. (**B**) PAF concentration in supernatants of HRCM derived from NCMs determined by ELISA (n=3). ****P* < 0.001 compared to 0 h group. (**C**) NCMs underwent hypoxia for 6 h, followed by reperfusion at indicated time points. Cell viability was determined by ATP assay. **P* < 0.05, ****P* < 0.001 compared to ctrl group. (**D and E**) NCMs were subjected into hypoxia for indicated time points, cell viability (**D**) and PAF concentration in supernatants (**E**) were determined (n=3). (**F**) PAF concentration in cardiac tissues (n=5). (**G**) Illustration of PAF metabolism, including *De novo* pathway and remodeling pathway. (**H**) Cardiac mRNA expression of *Pla2g4, Pla2g6, Pla2g7, Lpcat2, Chpt1, Pafah1b1* and *Pafah1b2*, normalized to *Actb* (n=5). ***P* < 0.01 compared to sham group. (**I**) Relative mRNA expression of *Pla2g4, Pla2g6, Pla2g7, Lpcat2, Chpt1, Pafah1b1* and *Pafah1b2*, normalized to *Actb* in cardiomyocytes underwent Hypoxia/Reoxygenation (H/R) (n=3). ****P* < 0.001 compared to mock group. (**J**) Primary cardiomyocytes were pretreated with 5 μM S-BEL, 0.5 μM darapladib or 0.5 μM pyrrophenone for 3 h, followed by H/R induction. PAF concentration in supernatants were determined (n=3). **P* < 0.05, ***P* < 0.01 compared to H/R group. (**K and L**) Primary cardiomyocytes were pretreated with 5 μM S-BEL for 3 h, followed by H/R induction. Ctrl CM and HRCM were collected. Representative images (**K**) with staining for MPO (green), H3Cit (red) and Hoechst (blue) and percentage of H3Cit positive cells (**L**) were shown (n=3). Scale bar, 50 μm. ***P* < 0.01, ****P* < 0.001 compared to HRCM group. (**M**) WT and *Gsdmd^-^*^/-^ mice were pre-treated with 10 mg/kg S-BEL 12 h before LAD ligation, followed by reperfusion for 24 h. Representative images of HE staining (upper panel), Masson’s trichrome staining (middle panel) of heart section and TTC/Evans Blue staining were shown. Red dotted lines indicate infarct area. Scale bar, 100 μm. (**N**) Percentage of infarct size within area at risk.

(**O, P**) serum dsDNA (**O**) and MPO-DNA nucleosome complex (**P**) were determined (n=4-5 mice per group). Data are shown as mean±SEM. **P* < 0.05, ***P* < 0.01 compared toWT-MI/R group. n.s., not significant.


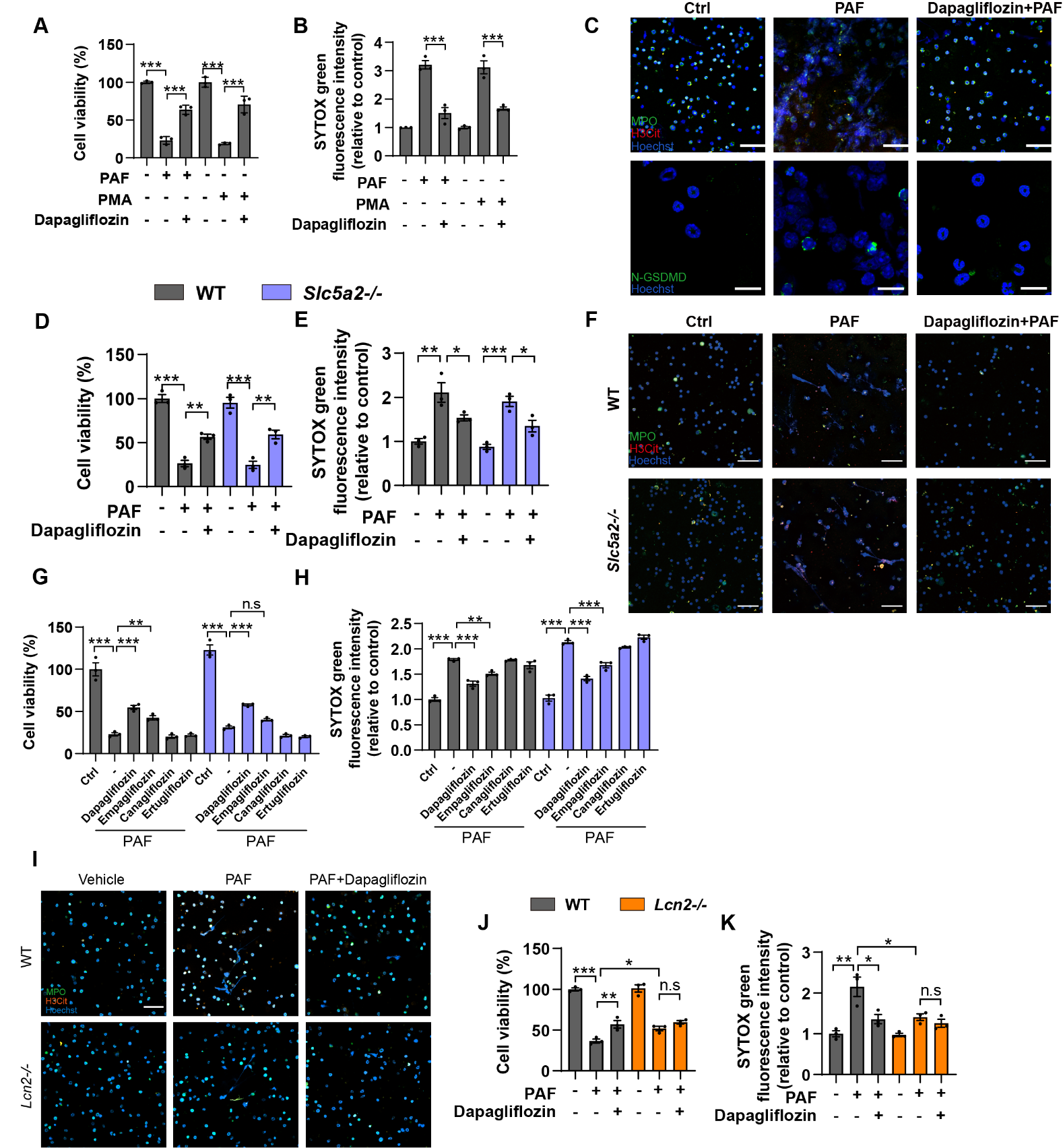


**Fig. S10. Dapagliflozin inhibits NETs formation independent of SGLT2.** (**A and B**) Primary neutrophils were pretreated with 20 μM dapagliflozin for 1 h followed by induction of NETosis using 5 μM PAF or 100 nM PMA for 3 h. ATP-based cell viability was determined (**A**). Sytox green-based NETosis assay (**B**) were shown (n=3). (**C**) Primary neutrophils were pretreated with 20 μM dapagliflozin for 1 h followed by induction of NETosis using 5 μM for 1 h. Representative images with staining for MPO (green), H3Cit (red) and Hoechst (blue) (upper panel, scale bar, 50 μm); N-GSDMD (green) and Hoechst (blue) (lower panel, scale bar, 20 μm) were shown. ****P* < 0.001 compared to PAF/PMA group. (**D, E**) Primary neutrophils were isolated from wild type (WT) or *Slc5a2^-^*^/-^ mice and pretreated with 20 μM dapagliflozin for 1 h, followed by induction of NETosis using 5 μM PAF or 100 nM PMA for 3 h. ATP-based cell viability was determined (**D**). Sytox green-based NETosis assay (**E**) were shown (n=3). (**F**) WT or *Slc5a2^-^*^/-^ neutrophils were pretreated with 20 μM dapagliflozin for 1 h followed by induction of NETosis using 5 μM PAF for 1 h. Representative images with staining for MPO (green), H3Cit (red) and Hoechst (blue) (upper panel, scale bar, 50 μm); N-GSDMD (green) and Hoechst (blue) (lower panel, scale bar, 20 μm) were shown. (**G and H**) WT and *Slc5a2*^-/-^ neutrophils were pretreated with 20 μM dapagliflozin, empagliflozin, canagliflozin and ertugliflozin for 1 h followed by induction of NETosis using 5 μM PAF for 3 h. ATP-based cell viability was determined (**G**). Sytox green-based NETosis assay (**H**) were shown (n=3). (**I**) Primary neutrophils isolated from WT and *Lcn2*^-/-^ neutrophils were pretreated with 20 μM dapagliflozin for 1 h followed by induction of NETosis using 5 μM for 3 h. Representative images with staining for MPO (green), H3Cit (red) and Hoechst (blue) were shown. Scale bar, 50 μm. (**J, K**) ATP-based cell viability was determined (**J**) and Sytox green-based NETosis assay (**K**) were shown (n=3). Data are shown as mean±SEM. **P* < 0.05, ***P* < 0.01, ****P* < 0.001 compared to PAF group. n.s., not significant.


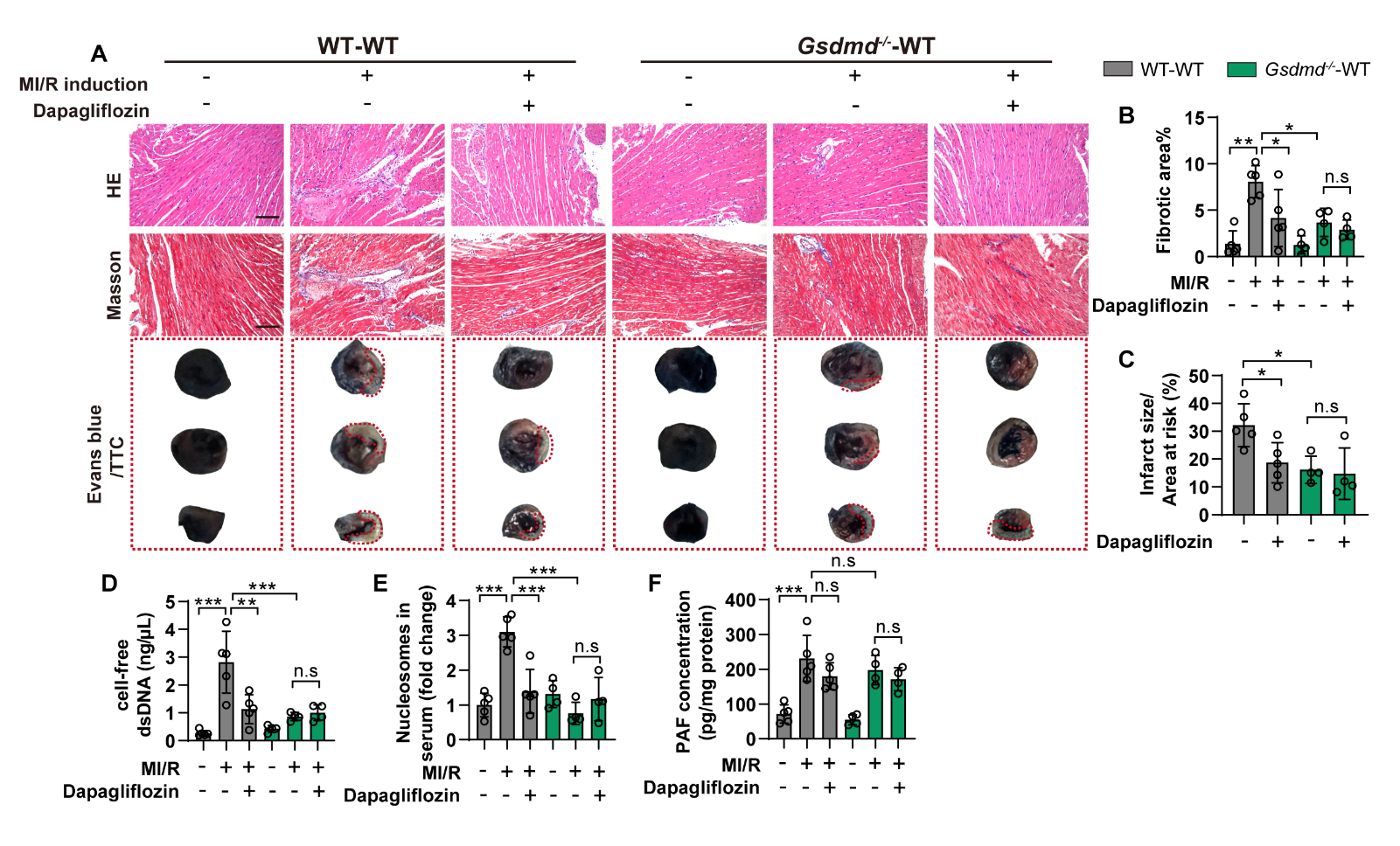


**Fig. S11. Dapagliflozin alleviate MI/R injury in a GSDMD-dependent manner in mice.** CD45.1 mice were treated with Ly6G neutralizing antibody and received either CD45.2 WT neutrophils or CD45.2 *Gsdmd*^-/-^ neutrophils, 50 mg/kg dapagliflozin were administered intraperitoneally 12 h before MI/R induction, then subjected into MI/R surgery. (**A**) Representative image of HE staining (upper panel, scale bar, 100 μm), Masson’s trichrome staining (middle panel, scale bar, 100 μm) and TTC/Evans Blue staining (lower panel) were shown. (**B**) Percentage of fibrotic area in each group, quantified data from (A). (**C**) Measurement of infarct size. (**D and E**) Cell-free dsDNA (**D**) and MPO-DNA nucleosome quantification (**E**) in serum. (**F**) Cardiac concentration of PAF determined by ELISA. n=4-5 mice per group. Data are shown as mean±SEM. **P* < 0.05, ***P* < 0.01, ****P* < 0.001 compared to WT-WT MI/R group, n.s., not significant compared to *Gsdmd*^-/-^-WT MIR group.


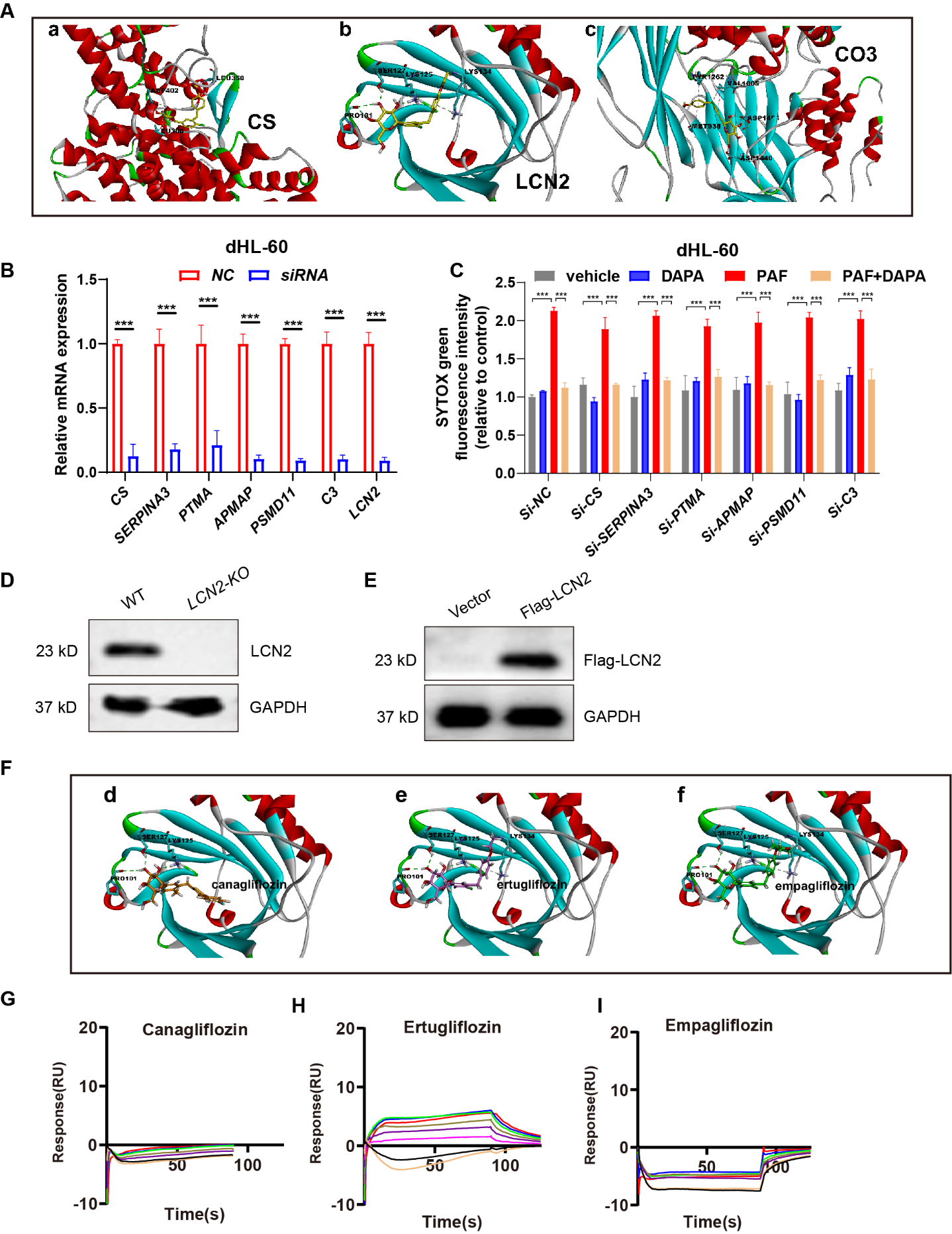


**Fig. S12. Identification of potential dapagliflozin target.** (**A**) Binding mode of dapagliflozin (yellow stick model) with three targets. (**a**) CS, (**b**) LCN2, and (**c**) CO3. (**B**) Relative expression of each gene after siRNA silencing. (**C**) dHL-60 cells were transfected with indicated siRNAs. Cells were pre-treated with 20 μM dapagliflozin for 1 h, followed by induction of NETosis by 20 μM PAF for another 3 h. Sytox green based cell death were determined (n=3). (**D**) Western blot analysis of HL-60 cells transfected with NGAL CRISPR/Cas9 KO plasmid. (**E**) Western blot analysis of HL-60 cells transfected with pCMV-LCN2(human)-FLAG-SV40-Neo plasmid. (**F**) Binding mode of three drugs targeting LCN2. (**d**) canagliflozin, (**e**) ertugliflozin and (**f**) empagliflozin. (**G to I**) Binding affinities of canagliflozin (**G**), ertugliflozin (**H**) and empagliflozin (**I**) to recombinant LCN2 protein were detected by SPR assay; equilibrium dissociation constant (KD) is indicated. Data are shown as mean±SEM. ****P* < 0.001 compared to PAF group.

**Table S1 TOP20 up-regulated and down-regulated proteins in cardiac tissues of MI/R mice compared to sham mice.**

|  | **Protein Name (Accession)** | **Fold change**  **MIR/Sham** | **Description** |
| --- | --- | --- | --- |
| **Up-regulated proteins** | | | |
| 1 | Saa1 (P05366) | 73.28749 | Serum amyloid A-1 protein, major acute phase protein |
| 2 | Saa2 (P05367) | 67.57231 | Serum amyloid A-2 protein, major acute phase reactant |
| 3 | Myl1 (E9PWG4) | 26.69095 | Myosin, light polypeptide 1 |
| 4 | Lcn2 (P11672) | 20.80134 | Neutrophil gelatinase-associated lipocalin, iron-trafficking protein involved in multiple processes |
| 5 | S100a8 (P27005) | 20.78433 | Protein S100-A8, regulation of inflammatory processes and immune response |
| 6 | Ighg3 (A0A1Y7VJN6) | 19.44236 | Immunoglobulin heavy constant gamma 3 |
| 7 | Plg (P20918) | 17.81313 | Plasminogen, dissolves the fibrin of blood clots |
| 8 | Lyz2 (P08905) | 17.32586 | Lysozyme C-2, active against bacteria |
| 9 | Apoc1 (P34928) | 16.97301 | Apolipoprotein C-I, Inhibitor of LDLR and VLDLR |
| 10 | S100a9 (P31725) | 16.50809 | Protein S100-A9, calcium- and zinc-binding protein, regulation of inflammatory processes and immune response |
| 11 | Ngp (O08692) | 13.05637 | Neutrophilic granule protein, inhibitor of cathepsin B (CTSB) activity |
| 12 | C4b (P01029) | 12.31659 | Complement C4-B, essential for the propagation of complement pathway |
| 13 | Vtn (P29788) | 12.0706 | Vitronectin, cell adhesion and spreading factor |
| 14 | Apoh (Q01339) | 10.97608 | Beta-2-glycoprotein 1, prevent activation of the intrinsic blood coagulation |
| 15 | Cfh (P06909) | 10.69054 | Complement factor H, Glycoprotein, modulating complement activation |
| 16 | Clu (Q06890) | 9.981293 | Clusterin, prevents aggregation of non native proteins |
| 17 | Chil3 (O35744) | 9.927979 | Chitinase-like protein 3, binds saccharides |
| 18 | Igkv4-53 (A0A075B677) | 9.258599 | Immunoglobulin kappa variable 4-53 |
| 19 | Camp (P51437) | 8.957248 | Cathelicidin antimicrobial peptide, Antimicrobial protein, binds to LPS |
| 20 | Lcp1 (Q61233) | 7.65671 | Plastin-2, Actin-binding protein, activation of T-cells |
| **Down-regulated proteins** | | | |
| 21 | Mavs (Q8VCF0) | 0.146502 | Mitochondrial antiviral-signaling protein, Adapter required for innate immune defense against viruses |
| 22 | Serpinb6b (O08804) | 0.151842 | Inhibits GzmA function |
| 23 | Phpt1 (Q9DAK9) | 0.160171 | Exhibits phosphohistidine phosphatase activity |
| 24 | Ptgr2 (D6RGL6) | 0.165454 | 15-oxoprostaglandin 13-reductase |
| 25 | Ybx1 (P62960) | 0.207151 | Y-box-binding protein 1, acts as a RNA-binding protein |
| 26 | Celf2 (S4R1S7) | 0.213808 | CUGBP, Elav-like family member 2 |
| 27 | Nqo1 (Q64669) | 0.217762 | NAD(P)H dehydrogenase [quinone] 1, catalyzes two-electron reduction of quinones to hydroquinones |
| 28 | Asrgl1 (Q8C0M9) | 0.24532 | Isoaspartyl peptidase/L-asparaginase, L-asparaginase and beta-aspartyl peptidase activity |
| 29 | Isoc1 (A0A494B952) | 0.250741 | Isochorismatase domain containing 1 |
| 30 | Perm1 (Q149B8) | 0.273333 | Regulates the expression of selective PPARGC1A/B and ESRRA/B/G target genes |
| 31 | Gyg1 (Q9R062) | 0.275207 | Glycogenin-1, Self-glucosylates |
| 32 | Otub1 (D3YWF6) | 0.276419 | ubiquitinyl hydrolase 1, Thiol-dependent hydrolysis of ester, thioester, amide, peptide and isopeptide bonds |
| 33 | Hspb8 (Q9JK92) | 0.281483 | Heat shock protein beta-8, displays temperature-dependent chaperone activity. |
| 34 | Rnh1 (A0A1B0GSG5) | 0.302211 | Ribonuclease inhibitor, inhibits RNASE1, RNASE2 and ANG |
| 35 | Vwa5a (Q99KC8) | 0.316292 | von Willebrand factor A domain-containing protein 5A, play a role in tumorigenesis as a tumor suppressor |
| 36 | Pgm1 (Q9D0F9) | 0.320084 | Phosphoglucomutase-1, participates in breakdown and synthesis of glucose |
| 37 | Nampt (Q99KQ4) | 0.342158 | Nicotinamide phosphoribosyltransferase, with immunomodulating anti-diabetic properties |
| 38 | Aimp1 (Q3UZG4) | 0.351048 | Aminoacyl tRNA synthetase complex-interacting multifunctional protein 1 |
| 39 | Txndc17 (Q9CQM5) | 0.36104 | Thioredoxin domain-containing protein 17, disulfide reductase, modulates TNF-alpha signaling and NF-kappa-B activation |
| 40 | Ppa1 (Q9D819) | 0.369156 | Inorganic pyrophosphatase |

**Table S2 Top20 endogenous metabolites in activating NETosis.**

| No | | | Name | SYTOX green fluorescence intensity  (Fold change relative to vehicle ctrl) | | | Description | |
| --- | --- | --- | --- | --- | --- | --- | --- | --- |
| 1 | | | Alloepipregnanolone | 8.626553087 | | | Allosteric modulator of GABA_A_R, involved in mood disorders | |
| 2 | | | Glycocholic acid | 8.167009015 | | | Bile acid with anticancer activity, targeting against pump resistance-related and non-pump resistance-related pathways | |
| 3 | | | Umbelliferone | 6.570067329 | | | A natural product of the coumarin family, is a fluorescing compound which can be used as a sunscreen agent | |
| 4 | | | Linoleic acid | 6.042029928 | | | A part of membrane phospholipids | |
| 5 | | | Palmitoleic acid | 5.764402095 | | | A composition of fatty acid | |
| 6 | | | Gamma- Linolenic acid | 5.4359194 | | | Involved in lipid metabolism | |
| 7 | | | 2-Phenylpropionic acid | 5.366940545 | | | An intermediate in alpha-Methylstyrene metabolism. | |
| 8 | | | 2'-Deoxyinosine | 5.34297615 | | | Keto acid in peripheral blood | |
| 9 | | | Arachidonic acid | 5.160930392 | | | Involved in purine nucleoside phosphorylase (PNP) deficiency | |
| 10 | | | Adenosine 5'-diphosphate | 4.861063563 | | | ADP, nucleoside diphosphate, the product of ATP dephosphorylation by ATPases | |
| 11 | | | 3-Hydroxyanthranilic acid | 4.705865571 | | | A tryptophan metabolite in the kynurenine pathway | |
| 12 | | | Adipic acid | 4.377439233 | | | Associated with HMG-CoA lyase deficiency, malonyl-Coa decarboxylase deficiency, and medium Chain acyl-CoA dehydrogenase deficiency | |
| 13 | | | Glycochenodeoxycholic acid | 4.344003195 | | | Immune regulation, induces apoptosis | |
| 14 | | | Farnesol | | 4.119305346 | | | Anti-microbial |
| 15 | | Thymidine | | | | 4.017174484 | | DNA synthesis inhibitor |
| 16 | | (S)-Leucic acid | | | | 3.203213551 | | Amino acid metabolite |
| 17 | | C16 PAF | | | | 2.475693256 | | Platelet activating factor, inflammatory mediator |
| 18 | 3-Phenyllactic acid | | | | | 2.226553087 | | Broad-spectrum antimicrobial compound |
| 19 | Taurochenodeoxycholic acid | | | | | 2.210137425 | | Anti-inflammatory and immune regulation |
| 20 | | | Hexadecanedioic acid | | 2.177781582 | | | Covalently linked to Sepharose 4B |

**Table S3 Candidate endogenous metabolites which induce NETs formation**

| No | Name | Percentage of H3Cit and MPO double positive cells | Description |
| --- | --- | --- | --- |
| 1 | C16 PAF | 54.22 | Platelet activating factor, inflammatory mediator |
| 2 | Gamma- Linolenic acid | 48.51 | Involved in lipid metabolism |
| 3 | Alloepipregnanolone | 42.12 | Allosteric modulator of GABA_A_R, involved in mood disorders |
| 4 | 3-Phenyllactic acid | 29.32 | Broad-spectrum antimicrobial compound |
| 5 | Uric acid | 28.97 | Antioxidant activity |
| 6 | Glycochenodeoxycholic acid | 22.30 | Immune regulation, induces apoptosis |
| 7 | 2-Oxovaleric acid | 22.08 | Keto acid in peripheral blood |

**Table S4 Candidate FDA-approved drugs with NETosis inhibition potential.**

|  |  | **% of cell death rate (PMA or PAF as 100% cell death)** | |  |
| --- | --- | --- | --- | --- |
| **No.** | **Compound Name** | **PAF** | **PMA** | **Targets** |
| **Anti-cancer drugs** | | | | |
| 1 | Mitoxantrone | -14.53 | -8.27 | Topoisomerase |
| 2 | Pirarubicin | -10.98 | -3.39 | Topoisomerase |
| 3 | Actinomycin D | -6.25 | -6.11 | DNA repair |
| 4 | Mitoxantrone (dihydrochloride) | -5.73 | -4.87 | Topoisomerase |
| 5 | Epirubicin (hydrochloride) | -5.15 | -5.24 | Topoisomerase |
| 6 | Doxorubicin (hydrochloride) | -14.79 | -5.10 | Topoisomerase |
| 7 | Suramin (sodium salt) | -2.32 | -3.63 | P2Y receptor，Topoisomerase |
| 8 | Pixantrone (dimaleate) | -4.74 | 13.28 | Topoisomerase |
| 9 | Pentostatin | 24.29 | -5.74 | Adenosine Deaminase |
| 10 | Zoledronic acid (monohydrate) | 20.70 | 0.68 | PKC |
| 11 | Dacarbazine | 22.46 | 9.56 | Nucleoside Antimetabolite |
| 12 | LEE011 (succinate hydrate) | 43.14 | -0.40 | CDK4/6 |
| 13 | Brigatinib | 17.97 | 39.15 | ALK |
| 14 | 5-Azacytidine | 24.88 | 38.04 | DNA Methyltransferase |
| 15 | Cyclophosphamide | 17.56 | 46.55 | DNA Alkylator/Crosslinker |
| 16 | Ifosfamide | 22.68 | 48.57 | DNA Alkylator/Crosslinker |
| 17 | Capsaicin | 32.54 | 42.43 | TRPV1 |
| 18 | Buserelin (Acetate) | 49.62 | 37.64 | GNRH Receptor |
| **Antibiotics/anti-virus drugs** | | | | |
| 1 | 1-Docosanol | -5.72 | -3.27 | HSV |
| 2 | Rimantadine (hydrochloride) | 14.95 | 18.97 | Influenza Virus |
| 3 | Zidovudine | 31.79 | 3.15 | HIV |
| 4 | Darunavir (Ethanolate) | 49.74 | -1.31 | HIV Protease |
| 5 | Natamycin | 26.06 | 11.73 | Fungal |
| 6 | Kanamycin (sulfate) | 41.14 | -1.14 | bacteria |
| 7 | Nadifloxacin | 37.74 | 10.50 | bacterial |
| 8 | Ribostamycin (sulfate) | 23.86 | 30.80 | bacterial |
| 9 | Furagin | 19.55 | 36.03 | bacterial |
| 10 | Amikacin (sulfate) | 20.30 | 37.82 | bacterial |
| 11 | Ofloxacin | 30.36 | 30.45 | bacterial |
| 12 | Cefixime | 36.47 | 24.79 | bacterial |
| 13 | Avibactam (sodium) | 28.82 | 35.30 | bacterial |
| 14 | Fusidic acid (sodium salt) | 41.86 | 25.40 | bacterial |
| 15 | Flucytosine | 48.81 | 22.59 | fungal |
| 16 | Kasugamycin (hydrochloride hydrate) | 33.40 | 38.73 | bacterial |
| 17 | Cefotiam (hydrochloride) | 36.33 | 37.47 | bacterial |
| 18 | Neomycin (sulfate) | 46.22 | 31.15 | Calcium Channel; Bacterial |
| 19 | p-Aminosalicylic acid (sodium salt dihydrate) | 36.56 | 40.95 | bacterial |
| 20 | Vidarabine | 42.96 | 34.75 | DNA/RNA Synthesis |
| 21 | Tenofovir (Disoproxil) | 49.56 | 28.98 | nucleotide reverse transcriptase |
| 22 | Nitroxoline | -1.80 | 19.06 | biofilm matrix |
| 23 | Nitazoxanide | 48.22 | 38.50 | Influenza Virus |
| **Anti-inflammation drugs** | | | | |
| 1 | Aspirin | -2.61 | -9.92 | COX |
| 2 | Sulindac | 23.32 | 18.51 | COX |
| 3 | Sulfasalazine | 46.33 | -2.55 | NF-κB |
| 4 | Pranlukast | 29.90 | 17.27 | Leukotriene Receptor，CYSLTR1 |
| 5 | Loxoprofen | 42.01 | 6.89 | COX |
| 6 | Dexchlorpheniramine (maleate) | 39.78 | 17.62 | Histamine Receptor |
| 7 | Bilastine | 26.08 | 44.75 | Histamine Receptor |
| 8 | Terbutaline (sulfate) | 31.14 | 41.58 | Adrenergic Receptor |
| 9 | Balsalazide | 37.87 | 36.17 | STAT3 |
| 10 | Acetaminophen | 36.65 | 40.40 | COX |
| 11 | Ibudilast | 40.70 | 43.63 | PDE |
| **Cardiovascular diseases related drugs** | | | | |
| 1 | Moexipril (hydrochloride) | 1.62 | 14.56 | ACE |
| 2 | Prostaglandin E2 | 23.04 | 13.91 | Prostaglandin Receptor |
| 3 | Macitentan | 29.24 | 9.10 | Endothelin Receptor |
| 4 | Torsemide | 18.46 | 21.53 | Na+/2Cl-/K+ cotransporter carrier protein |
| 5 | Tiotropium (Bromide) | 21.23 | 24.14 | mAChR |
| 6 | Naftidrofuryl (oxalate) | 8.28 | 44.77 | 5-HT2 |
| 7 | Eprosartan (mesylate) | 39.19 | 14.54 | Ang II |
| 8 | Dexrazoxane (Hydrochloride) | 26.43 | 29.82 | iron |
| 9 | Vernakalant (Hydrochloride) | 38.59 | 22.18 | Potassium Channel |
| 10 | Fondaparinux (sodium) | 16.66 | 47.93 | Factor Xa |
| 11 | Riociguat | 42.47 | 30.27 | Guanylate Cyclase |
| 12 | Gliclazide | 45.63 | 29.46 | Potassium Channel |
| 13 | Pyrithioxin (dihydrochloride) | 49.27 | 27.29 | glucose metabolism |
| 14 | L-(-)-α-Methyldopa (hydrate) | 44.82 | 37.14 | α2-adrenergic receptor |
| 15 | Ozagrel | 46.72 | 40.08 | Factor Xa |
| 16 | Temocapril (hydrochloride) | 44.01 | 45.87 | ACE |
| 17 | Alfuzosin | 44.54 | 48.26 | α1 adrenergic receptor |
| **Metabolic regulation drugs** | | | | |
| 1 | Dapagliflozin | 16.14 | -3.76 | SGLT2 |
| 2 | Ciprofibrate | 23.42 | 12.53 | PPAR |
| 3 | Acetohexamide | 14.37 | 26.06 | NA |
| 4 | Lorcaserin (Hydrochloride) | -4.34 | 48.20 | 5-HT Receptor |
| 5 | Propylthiouracil | 40.07 | 9.94 | thyroperoxidase， 5'-deiodinase inhibitor |
| 6 | Metyrapone | 31.53 | 19.20 | CYP11B1 |
| 7 | Rosuvastatin (Calcium) | 19.48 | 33.19 | HMG-CoA reductase |
| 8 | Methylthiouracil | 42.33 | 13.43 | NF-κB |
| 9 | Deoxycholic acid | 31.52 | 31.39 | TGR5/FXR |
| 10 | Febuxostat | 22.65 | 42.58 | Xanthine Oxidase |
| 11 | Probucol | 36.98 | 32.26 | VLDL cholesterol |
| 12 | Nateglinide | 28.73 | 48.84 | DPP IV |
| 13 | Clinofibrate | 32.60 | 48.24 | HMG-CoA reductase |
| **Neurological diseases related drugs** | | | | |
| 1 | Levobetaxolol (hydrochloride) | 23.28 | 11.16 | Adrenergic Receptor |
| 2 | Entacapone | 33.08 | 3.39 | catechol-O-methyltransferase |
| 3 | Dolasetron | 37.40 | 1.20 | 5-HT Receptor |
| 4 | Bupropion (hydrochloride) | 18.30 | 22.03 | Dopamine Transporter |
| 5 | Homatropine (methylbromide) | 37.63 | 6.19 | AChR |
| 6 | Fosphenytoin (disodium) | 41.20 | 12.34 | NA |
| 7 | Zonisamide | 34.451 | 19.46 | Calcium channel inhibitor |
| 8 | Diphenmanil (methylsulfate) | 46.72 | 8.78 | mAChR |
| 9 | Aceglutamide | 49.08 | 7.78 | glutamine |
| 10 | Primidone | 29.83 | 41.36 | GABA receptor |
| 11 | Amisulpride | 49.27 | 37.76 | Dopamine Receptor |
| 12 | Adiphenine (hydrochloride) | 47.20 | 34.57 | nAChR |
| **Others** | | | | |
| 1 | Methylene Blue | -3.59 | -1.57 | NA |
| 2 | Azelaic acid | 0.77 | -2.11 | NA |
| 3 | Sodium Fluoride | 32.03 | -2.99 | NA |
| 4 | Dithranol | 29.62 | 1.78 | DNA repair |
| 5 | Halcinonide | 15.88 | 15.78 | Smo |
| 6 | Eflornithine (hydrochloride, hydrate) | -5.93 | 38.31 | ornithine decarboxylase |
| 7 | Chlorophyllin (sodium copper salt) | 12.67 | 21.18 | NA |
| 8 | Prilocaine | 34.13 | 21.96 | Na+/K+ ATPase |
| 9 | Benzyl alcohol | 38.73 | 22.05 |  |
| 10 | Risedronate (sodium) | 43.78 | 22.85 | bone resorption |
| 11 | Benzyl benzoate | 43.52 | 24.91 | NA |
| 12 | L-Epinephrine | 46.70 | 22.00 | Adrenergic Receptor |
| 13 | Lifitegrast | 43.14 | 29.53 | Integrin |
| 14 | Phentolamine (mesylate) | 35.33 | 38.10 | Adrenergic Receptor |
| 15 | Prucalopride | 34.23 | 47.20 | 5-HT4 |
| 16 | Fasudil (Hydrochloride) | 34.18 | 47.38 | PKC, ROCK |
| 17 | Pralidoxime (chloride) | 39.51 | 46.44 | AChE |
| 18 | Ibandronate (Sodium Monohydrate) | 41.77 | 45.59 | NA |
| 19 | Omeprazole | 43.25 | 46.65 | proton pump |
| 20 | Alverine (citrate) | 49.60 | 40.83 | 5-HT1A |
| 21 | Norethindrone acetate | 42.68 | 48.86 | Progesterone Receptor |
| 22 | Vigabatrin | 47.18 | 44.85 | GABA receptor |

**Table S5 Normalized peptide intensity of 35 candidate peptides between Dapa group and Ctrl group.**

|  | Protein Name | Vehicle-1 | Vehicle-2 | Vehicle-3 | Dapa-1 | Dapa-2 | Dapa-3 |
| --- | --- | --- | --- | --- | --- | --- | --- |
| 1 | TPR | 0.031863 | 0.032675 | 0.030489 | 0.031675 | 0.055917 | 0.052334 |
| 2 | RL8 | 0.050964 | 0.051352 | 0.053129 | 0.051815 | 0.087536 | 0.081438 |
| 3 | RL31 | 0.103695 | 0.092778 | 0.104883 | 0.100452 | 0.158004 | 0.160775 |
| 4 | RL24 | 0.01324 | 0.0124 | 0.012877 | 0.012839 | 0.035883 | 0.032247 |
| 5 | Lamin B1 | 0.000419 | 0.000353 | 0.000424 | 0.000399 | 0.001029 | 0.001071 |
| 6 | Lamin B2 | 0.115622 | 0.089826 | 0.09832 | 0.101256 | 0.256617 | 0.25264 |
| 7 | LAP2B | 0.06876 | 0.085869 | 0.089638 | 0.081422 | 0.15867 | 0.158424 |
| 8 | HSP7C[610-646] | 0.002766 | 0.00265 | 0.002362 | 0.002593 | 0.0045 | 0.00427 |
| 9 | HNRPD | 0.012631 | 0.012404 | 0.013952 | 0.012996 | 0.025997 | 0.027143 |
| 10 | H2A1G | 0.00204 | 0.001566 | 0.00207 | 0.001892 | 0.004017 | 0.003802 |
| 11 | MICA1 | 0.062094 | 0.056047 | 0.057203 | 0.058448 | 0.117115 | 0.136445 |
| 12 | Filamin-A[705-724] | 0.000277 | 0.000262 | 0.000309 | 0.000283 | 0.000542 | 0.000526 |
| 13 | Filamin-A[2491-2500] | 0.001239 | 0.001124 | 0.001239 | 0.001201 | 0.001765 | 0.001928 |
| 14 | Filamin-B | 0.032557 | 0.039664 | 0.037982 | 0.036734 | 0.072985 | 0.073645 |
| 15 | VASP | 0.006796 | 0.007012 | 0.006734 | 0.006848 | 0.013055 | 0.012113 |
| 16 | MOES | 0.002467 | 0.002643 | 0.003211 | 0.002774 | 0.006133 | 0.005692 |
| 17 | CYRIB | 0.017601 | 0.016693 | 0.025267 | 0.019854 | 0.061962 | 0.063062 |
| 18 | COF1[38-45] | 0.000428 | 0.000506 | 0.000515 | 0.000483 | 0.001077 | 0.001112 |
| 19 | Cs | 0.163722 | 0.211783 | 0.214823 | 0.196776 | 0.58252 | 0.535155 |
| 20 | DC1L1 | 0.005804 | 0.005265 | 0.005506 | 0.005525 | 0.010206 | 0.01019 |
| 21 | GAPDH[225-240] | NA | 0.000456 | 0.000497 | 0.000476 | 0.000774 | 0.000776 |
| 22 | GNB2 | 0.023469 | 0.022217 | 0.025317 | 0.023667 | 0.03675 | 0.039274 |
| 23 | CAH2 | 0.043461 | 0.045668 | 0.037661 | 0.042263 | 0.069419 | 0.070175 |
| 24 | MDH1[301-310] | 0.088672 | 0.090816 | 0.099667 | 0.093051 | 0.177188 | 0.165804 |
| 25 | MDH1[300-310] | 0.102432 | 0.095554 | 0.105141 | 0.101042 | 0.195225 | 0.187302 |
| 26 | Serpina3k | 0.648797 | 0.491544 | 0.723443 | 0.621261 | 1.714261 | 2.009996 |
| 27 | MA2B1 | 0.019201 | 0.022337 | 0.021183 | 0.020907 | 0.039246 | 0.044938 |
| 28 | PTMA | 0.029435 | 0.050674 | 0.055432 | 0.04518 | 0.391829 | 0.381939 |
| 29 | MPO | 0.024418 | 0.023493 | 0.026308 | 0.02474 | 0.041725 | 0.038256 |
| 30 | LCN2 | 0.001507 | 0.001438 | 0.001435 | 0.00146 | 0.004669 | 0.005529 |
| 31 | APMAP | 0.040438 | 0.053996 | 0.045205 | 0.046546 | 0.758001 | 0.618479 |
| 32 | ACINU | 0.015763 | 0.016232 | 0.01498 | 0.015659 | 0.028667 | 0.031717 |
| 33 | PSMD7 | 0.152522 | 0.180971 | 0.195283 | 0.176259 | 0 | 0.656672 |
| 34 | RS3A | 0.005479 | 0.004922 | 0.005443 | 0.005281 | 0.017206 | 0.016285 |
| 35 | ROA2 | 0.023118 | 0.024966 | 0.023156 | 0.023747 | 0.045292 | 0.044014 |

**Table S6 Characteristics of the patient Population (n=72).**

|  | DM patients Pre-PCI (use dapagliflozin, n=7) | DM patients post-PCI (use dapagliflozin, n=7) | DM patients pre-PCI (no dapagliflozin, n=33) | DM patients post-PCI (no dapagliflozin, n=32) | Normal patients Pre-PCI (no dapagliflozin, n=32) | Normal patients Post-PCI (no dapagliflozin, n=32) |
| --- | --- | --- | --- | --- | --- | --- |
| characteristics |  | | | | | |
| sex [% (n)] |  |  |  |  |  |  |
| male | 57.1 (7) | 57.1 (7) | 75.8 (33) | 75.8 (33) | 93.8 (32) | 93.8 (32) |
| female | 42.9 (7) | 42.9 (7) | 24.2 (33) | 24.2 (33) | 6.2 (32) | 6.2 (32) |
| Age (years) | 62.4 (10.9) | 62.4 (10.9) | 63.9 (13.5) | 63.9 (13.5) | 59.0 (14.5) | 59.0 (14.5) |
| Smoking [% (n)] | 42.9 (7) | 42.9 (7) | 57.6 (33) | 57.6 (33) | 68.8 (32) | 68.8 (32) |
| Drinking [% (n)] | 28.6 | 28.6 | 42.4 | 42.4 | 56.2 (32) | 56.2 (32) |
| History of coronary artery disease [% (n)] | 0 (7) | 0 (7) | 9.1 (33) | 9.1 (33) | 3.1 (32) | 3.1 (32) |
| History of familial coronary artery disease [% (n)] | 28.6 (7) | 28.6 (7) | 15.2 (33) | 15.2 (33) | 25 (32) | 25 (32) |
| High blood pressure [% (n)] | 100 (7) | 100 (7) | 69.7 (33) | 69.7 (33) | 43.8 (32) | 43.8 (32) |
| Hyperlipidaemia [% (n)] | 28.6 (7) | 28.6 (7) | 27.3 (33) | 27.3 (33) | 40.6 (32) | 40.6 (32) |
| BMI (kg/m^2^) | 26.6 (4.1) | 26.6 (4.1) | 24.3 (7.1) | 24.3 (7.1) | 25.7 (3.0) | 25.7 (3.0) |
| Pre-antiplatelet treatment [% (n)] | 14.3 (7) | 14.3 (7) | 30.3 (33) | 30.3 (33) | 50 (32) | 50 (32) |
| Pre-anticoagulant treatment [% (n)] | 0 (7) | 0 (7) | 6.1 (33) | 6.1 (33) | 0 (32) | 0 (32) |
| Pre-statin treatment [% (n)] | 0 (7) | 0 (7) | 12.1 (33) | 12.1 (33) | 6.3 (32) | 6.3 (32) |
| Pre-β-blocker treatment [% (n)] | 28.6 (7) | 28.6 (7) | 12.1 (33) | 12.1 (33) | 3.1 (32) | 3.1 (32) |
| Pre-ACEI/ARB treatment [% (n)] | 57.1 (7) | 57.1 (7) | 21.2 (33) | 21.2 (33) | 9.4 (32) | 9.4 (32) |
| Pre-Nitrate treatmeent [% (n)] | 0 (7) | 0 (7) | 6.1 (33) | 6.1 (33) | 0 (32) | 0 (32) |
| IVSTd ({IVSTd}):#.## cm | 0.94 (0.19) | / | 0.90 (0.14) | / | 0.91 (0.13) | / |
| LVDd ({LVDd}):#.## cm | 5.37 (0.40) | / | 5.13 (0.85) | / | 5.27 (0.89) | / |
| LVPWTd ({LVPWTd}):#.## cm | 0.93 (0.14) | / | 0.86 (0.14) | / | 0.90 (0.08) | / |
| AoD ({AoD}):#.## cm | 3.42 (0.24) | / | 3.02 (0.61) | / | 3.25 (0.33) | / |
| LAD ({LAD}):#.## cm | 4.03 (0.40) | / | 3.92 (0.40) | / | 3.91 (0.30) | / |
| EF ({EF}):##.#% | 45.1 (10.4) | / | 45.9 (5.9) | / | 47.5 (5.3) | / |
| dsDNA (ng/μL) | 0.59 (0.08) | 0.83 (0.12) | 0.66 (0.11) | 0.85 (0.33) | 0.66 (0.13) | 0.93 (0.38) |
| S100A8/A9 (ng/mL) | 1047.0 (223.9) | 1455.0 (382.8) | 993.8 (263.3) | 1907.0 (347.9) | 923.2 (301.0) | 1840.0 (397.8) |
| PAF (ng/mL) | 1.22 (0.60) | 1.70 (0.58) | 1.28 (0.78) | 4.66 (5.32) | 1.37 (1.20) | 3.46 (4.03) |
| LCN2 (ng/mL) | 40.10 (15.67) | 45.31 (19.71) | 43.18 (16.86) | 63.95 (21.72) | 42.72 (21.13) | 56.55 (22.49) |
| ALT (U/L) | 20.29 (14.86) | 28.71 (13.70) | 30.61 (18.18) | 47.7 (26.58) | 25.3 (14.8) | 49.2 (24.2) |
| AST (U/L) | 39.57 (20.90) | 116.6 (81.96) | 79.58 (91.65) | 256.5 (218.0) | 78.7 (87.3) | 244.3 (169.8) |
| LDH (U/L) | 197.3 (50.98) | 425.0 (123.7) | 327.1 (209.2) | 771.9 (583.1) | 318.0 (185.1) | 731.8 (468.2) |
| CK (U/L) | 162.1 (183.2) | 1594 (690.5) | 700.7 (1040) | 2270.0 (2010.0) | 744.6 (961.4) | 2323.0 (1808.0) |
| CK-MB (U/L) | 20.23 (17.31) | 143.5 (93.20) | 48.75 (60.13) | 170.8 (136.1) | 72.3 (85.5) | 226.3 (169.7) |

Continuous variables are given as mean (standard deviation), and categorical variables are given as percentage (number). BMI, body mass index; IVSTd, interventricular septum thickness in diastole; LVDd, left ventricular diastolic diameter; LVPWTd, left ventricle posterior wall thickness in diastole; AoD, aortic diameter; LAD, left atrial diameter; EF, eject fraction; PAF, platelet activating factor; LCN2, lipocalin-2; ALT, alanine aminotransferase; AST, aspartate aminotransferase; LDH, lactate dehydrogenase; CK, creatine Kinase; CK-MB, creatine kinase-MB.

**Table S7** ***SiRNA* Sequences**

| No | Gene | Sense 5’ to 3’ | Antisense 5’ to 3’ |
| --- | --- | --- | --- |
| 1 | CS (homo-ID:1431) | GAGCAUAUGCACAGGGUAUTT | AUACCCUGUGCAUAUGCUCTT |
| 2 | SERPINA3 (homo-ID:12) | AAGACAAGAUGGAGGAAGUTT | ACUUCCUCCAUCUUGUCUUTT |
| 3 | PTMA (homo-ID:5757) | CUGAAGAUGAUGAGGAUGATT | UCAUCCUCAUCAUCUUCAGTT |
| 4 | APMAP (homo-ID:57136) | GCAGAAAGGCUGUUUGAAATT | UUUCAAACAGCCUUUCUGCTT |
| 5 | PSMD11(homo-ID:5717) | UGGCCAAGUUGUAUGAUAA | UUAUCAUACAACUUGGCCA |
| 6 | C3(homo-ID:718) | CGGAAAAGGAGGAUGGAAA | UUUCCAUCCUCCUUUUCCG |
| 7 | LCN2(homo-ID:3934) | CUUCGGAACUAAAGGAGAA | UUCUCCUUUAGUUCCGAAG |
| 8 | Si-NC | UUCUCCGAACGUGUCACGUTT | ACGUGACACGUUCGGAGAATT |

**Table S8 Primers for quantitative real-time PCR**

| No | Gene | Forward 5’ to 3’ | Reverse 5’ to 3’ |
| --- | --- | --- | --- |
| 1 | CS | GGTGGCATGAGAGGCATGAA | TAGCCTTGGGTAGCAGTTTCT |
| 2 | SERPINA3 | TGCCAGCGCACTCTTCATC | TGTCGTTCAGGTTATAGTCCCTC |
| 3 | PTMA | TCAGACGCAGCCGTAGACA | GCATTCCCGTTAGCAGGGG |
| 4 | APMAP | CATTGCCCGGTTTGGTTCG | CACTTCACGTTTCCAGGGATTTA |
| 5 | PSMD11 | GTGTTTAGAGTGCATCGAATGGG | AGAGCTTTGTCGTCCATCTTTTT |
| 6 | C3 | GGGGAGTCCCATGTACTCTATC | GGAAGTCGTGGACAGTAACAG |
| 7 | LCN2 | GACAACCAATTCCAGGGGAAG | GCATACATCTTTTGCGGGTCT |
| 8 | CS | GGTGGCATGAGAGGCATGAA | TAGCCTTGGGTAGCAGTTTCT |
| 9 | GAPDH | ACAACTTTGGTATCGTGGAAGG | GCCATCACGCCACAGTTTC |
| 10 | Pla2g6 | GCAAGCTGATTACCAGGAAGG | GAGAGAAGAGGGGGTGAGTTG |
| 11 | Pla2g7 | TCACAAGCTCCAATCGGTGAT | CGACGGGGTACGATCCATTTC |
| 12 | Pla2g4 | CAGCACATTATAGTGGAACACCA | AGTGTCCAGCATATCGCCAAA |
| 13 | Chpt1 | ACTGAGATCCAGGTAGCTTTAGT | GTAGACCCATTCTTGCCAACA |
| 14 | Lpcat2 | CCCTTCGTCCAGCAGACTAC | GCAGCAAAATTATTCCAACCAGT |
| 15 | Pafah1b1 | CCTTGGATTTCCATAAGACGGC | ACTCCCACACCTTTACTGTTTG |
| 16 | Pafah1b2 | GGTAGGGACAAACAACCACGA | AGACCCAGCACAATGATCTTG |
| 17 | Actb | GGCTGTATTCCCCTCCATCG | CCAGTTGGTAACAATGCCATGT |
